# Bromodomain-Driven Regulation of Stem Cells: A Potential Target for Cancer Therapeutic Intervention

**DOI:** 10.1007/s12015-025-11029-w

**Published:** 2025-12-09

**Authors:** Muthuvel Jothi, Anil Kumar Devakrishnan, Krishna Kumar Haridhasapavalan

**Affiliations:** 1 Graduate School of Agricultural Science, Osaka Metropolitan University, Osaka, 5998531 Japan; 2 Present Address: Department of Biotechnology, Periyar Maniammai Institute of Science & Technology (Deemed to be University), Tamil Nadu 613403 Thanjavur, India; 3 Department of Pathology and Immunology, Baylor College of Medicine, Houston, TX 77030 USA; 4 Department of Cell and Developmental Biology, Perelman School of Medicine, University of Pennsylvania, Philadelphia, PA 19104 USA

**Keywords:** Bromodomain, Histone acetylation, Bromodomain inhibitors, Epigenetic regulators, Cancer stem cells

## Abstract

All cells within an organism share identical genetic material, yet epigenetic mechanisms determine stem cell fate by precisely regulating transcriptional programs. Histone acetylation is a key epigenetic modification that establishes an open chromatin structure, which is recognized by proteins involved in modulating chromatin dynamics essential for stem cell functions. Bromodomain (BrD)-containing proteins specifically recognize acetylated lysines on histones and act as critical epigenetic regulators within larger protein complexes. This review comprehensively describes the BrD protein family, highlighting their structural classifications and diverse functions, and explores their critical roles in regulating stem cell pluripotency and differentiation, and their implications in cancer development. Dysregulated BrD proteins can drive cancer by increasing stem cell-like features and tumor heterogeneity, making them a potential target for cancer treatment. Furthermore, this review emphasizes BrD inhibitors as promising therapeutic targets capable of targeting cancer stem cells and potentially mitigating cancer progression. Understanding the detailed functions and regulatory pathways of BrD proteins may open new avenues for improved cancer stem cell-targeted therapies.

## Introduction

Molecular and cellular heterogeneity of cancer poses significant challenges to the advancement of personalized and precision medicines [1]. This heterogeneity is driven by the continuous evolution of tumors, which continues be acquiring new molecular traits even after malignant transformation [2]. These changes give rise to distinct subpopulations of cancer cells with unique characteristics and varying resistance to advanced treatments. Technological advances in molecular profiling have revealed that cancer progression is a nonlinear, often unpredictable process driven by the disruption of essential cellular pathways that promote uncontrolled growth, resist cell death, stimulate angiogenesis, and enable invasion and metastasis [1]. While current precision therapies are designed to target specific molecular characteristics, such as activated signaling pathways and impaired DNA damage repair mechanisms, improving patient outcomes demands a thorough understanding of the complex molecular landscape of cancer [3]. Achieving this requires a comprehensive, multi-layered approach by integrating multi-omics data; however, in this review, we primarily focused on epigenetic heterogeneity.

DNA methylation, post-translational modifications of histones, microRNAs, and long non-coding RNAs represent fundamental epigenetic mechanisms that regulate gene expression without altering the underlying DNA sequences [4]. These epigenetic modifications are heritable and dynamically influence chromatin structure and accessibility, thereby regulating critical nuclear processes, including transcriptional control, DNA replication fidelity, and DNA damage repair pathways [4]. Collectively, epigenetic regulation provides a sophisticated layer of governing gene activity, enabling cells with identical genetic material to differentiate into distinct cell types, adopt specialized functions, and effectively respond to environmental or developmental stimuli. Given the essential role of epigenetic mechanisms in maintaining normal stem cell identity, their dysregulation frequently contributes to the acquisition and maintenance of cancer stem cell (CSC)-like properties [5]. Notably, altered chromatin modifications, such as aberrant histone acetylation, can promote and stabilize the CSC state [5].

Lysine acetylation is a key post-translational modification that regulates gene transcription and DNA repair. Histone acetyltransferases (HATs) or lysine acetyltransferases (KATs) catalyze the addition of the acetyl group from acetyl-CoA to the amino group of lysine residues, typically located near the N-terminal tail of core histone proteins [6]. This acetylation reduces the positive charge on histones, thereby weakening their interaction with negatively charged DNA and resulting in an open chromatin structure, known as euchromatin, that is associated with active gene expression [7]. In contrast, histone deacetylases (HDACs) remove these acetyl groups, restoring histone-DNA affinity and leading to chromatin condensation into a transcriptionally inactive form called heterochromatin. The dynamic balance between acetylation and deacetylation, first observed in the mid-20th century, is essential for gene regulation [8]. In 1996, Allis and colleagues identified general control nonderepressible 5 (GCN5) as the first HAT, followed by Schreiber and colleagues discovering HDAC1 as the first HDAC, emphasizing the regulatory role of histone modifications [9–11].

Bromodomains (BrDs) are conserved protein domains that recognize and bind to acetylated lysine (Kac) residues, functioning as chromatin “readers” that help regulate gene expression by recruiting transcriptional complexes in response to various stimuli [12]. Originally identified in *Drosophila*, the brm gene was first characterized as a BrD-containing chromatin remodeler involved in transcriptional regulation. In addition to their roles in gene regulation, BrDs are found in proteins with enzymatic activities and in scaffolding components that organize large regulatory complexes [13, 14]. Due to their diverse functions, BrD-containing proteins have been implicated in a range of diseases, including cancer, cardiovascular, metabolic, and neurological disorders [13, 14]. The development of epigenetic inhibitors targeting BrDs have shown strong potential in preclinical cancer research [15]. Although early efforts focused on small molecule inhibitors of the BrD and extra-terminal (BET) family, recent studies have expanded to include compounds that selectively target non-BET BrDs, offering new therapeutic avenues and deeper insight into cancer biology [15]. These inhibitors have deepened our understanding of the functional roles of non-BET BrD proteins, while ongoing structural studies have revealed how BrD specifically recognizes histone modifications. This knowledge has enabled the design of next-generation inhibitors with improved selectivity for individual BrD proteins, including those outside the BET family [16]. Despite progress, the role of BrD proteins in cancer progression remains unclear, and further study of their expression, interactions, and regulatory functions may reveal key cancer-related mechanisms.

This review explores the pivotal role of BrD family of proteins in the regulation and maintenance of pluripotent, multipotent, and cancer stem cells. We highlighted specific BrD family members, including both BET and non-BET proteins, that are involved in stem cell regulation and discussed their emerging potential as promising therapeutic targets. Furthermore, we have discussed the development of selective small-molecule inhibitors that target BrD proteins in cancer therapy. Additionally, we explored combinatorial therapeutic strategies that incorporate BrD inhibitors with other epigenetic or chemotherapeutic agents, which may enhance efficacy and overcome resistance, offering a more comprehensive approach to eliminating CSCs that drive tumor progression. Thus, targeting BrDs holds significant promise for advancing personalized cancer therapies by enabling more precise and tailored treatment strategies across a wide range of tumor types.

## Basic Structural Features of BrDs

BrDs are approximately 110 amino acids in length and form a globular structure consisting of four left-handed antiparallel α-helices (αZ, αA, αB, and αC), connected by two interhelical loops, ZA and BC [13]. These loops form a hydrophobic pocket that stabilizes the four-helix bundle and serves as the binding site for Kac [14]. Variations in the length and amino acid composition of the ZA and BC loops influence Kac-binding specificity by interacting with adjacent residues.

**Fig. 1:**
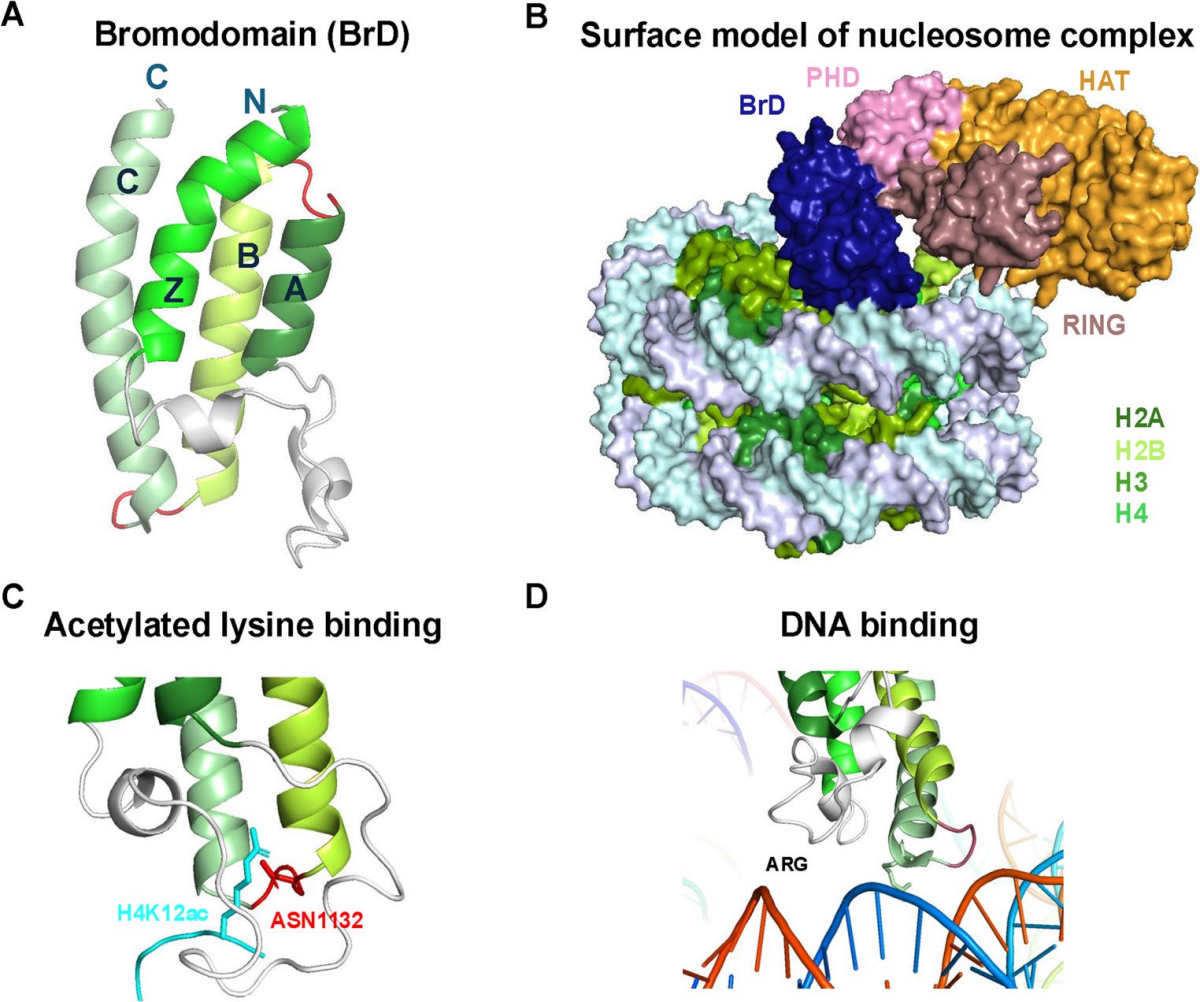
Structural insights into bromodomain (BrD) and its chromatin interactions. **(A)** The BrD structure of p300 (Protein Data Bank [PDB] identifier: 7W9V), with the conserved four α-helical regions colored and annotated. The BRD α-helices (Z, A, B, and C) are connected by loop regions (red). **(B)** Overall surface map of the p300 nucleosome complex (7W9V). The BrD (blue), PHD (pink), RING (brown), and HAT (orange) domains are highlighted. **(C)** BrD of p300 (PDB ID: 8HAG) bound to the H4-acetylated nucleosome (H4K12ac). The conserved ASN1132 residue (red) is located within 4 Å from H4Kac (cyan). **(D)** The p300 BrD (7W9V) bound to the minor groove of DNA

The first structural evidence of Kac recognition by BrDs came from the p300/CBP-associated factor (PCAF) BrD bound to acetyl-histamine (a small molecule mimic of Kac) [17], revealing the mechanism of BrD in recognizing Kac. Critical residues for Kac recognition across all BrDs include tyrosine and asparagine [13]. The asparagine residue in the BC loop forms two hydrogen bonds with the amide nitrogen and Kac carbonyl oxygen, along with an additional hydrogen bond to the sidechain OH of the conserved tyrosine residue [13, 14] (Fig. 1).

Among the 61 BrDs identified, 48 are classified as typical BrDs based on the presence of this conserved asparagine, while the remaining 13 are termed atypical BrDs (aBrDs) due to the alternate residues such as aspartic acid, tyrosine, or threonine, and exhibit low affinity to Kac binding [18]. The function of aBrDs likely relates to nearby protein domains and requires further investigation [13]. BrDs also exhibit conserved N-terminal domains and variable C-terminal domains, which support their role in protein-protein interactions [19]. Despite these structural differences, Kac recognition remains largely conserved across BrD proteins.

### Functional Classification of BrD Proteins

**Fig. 2:**
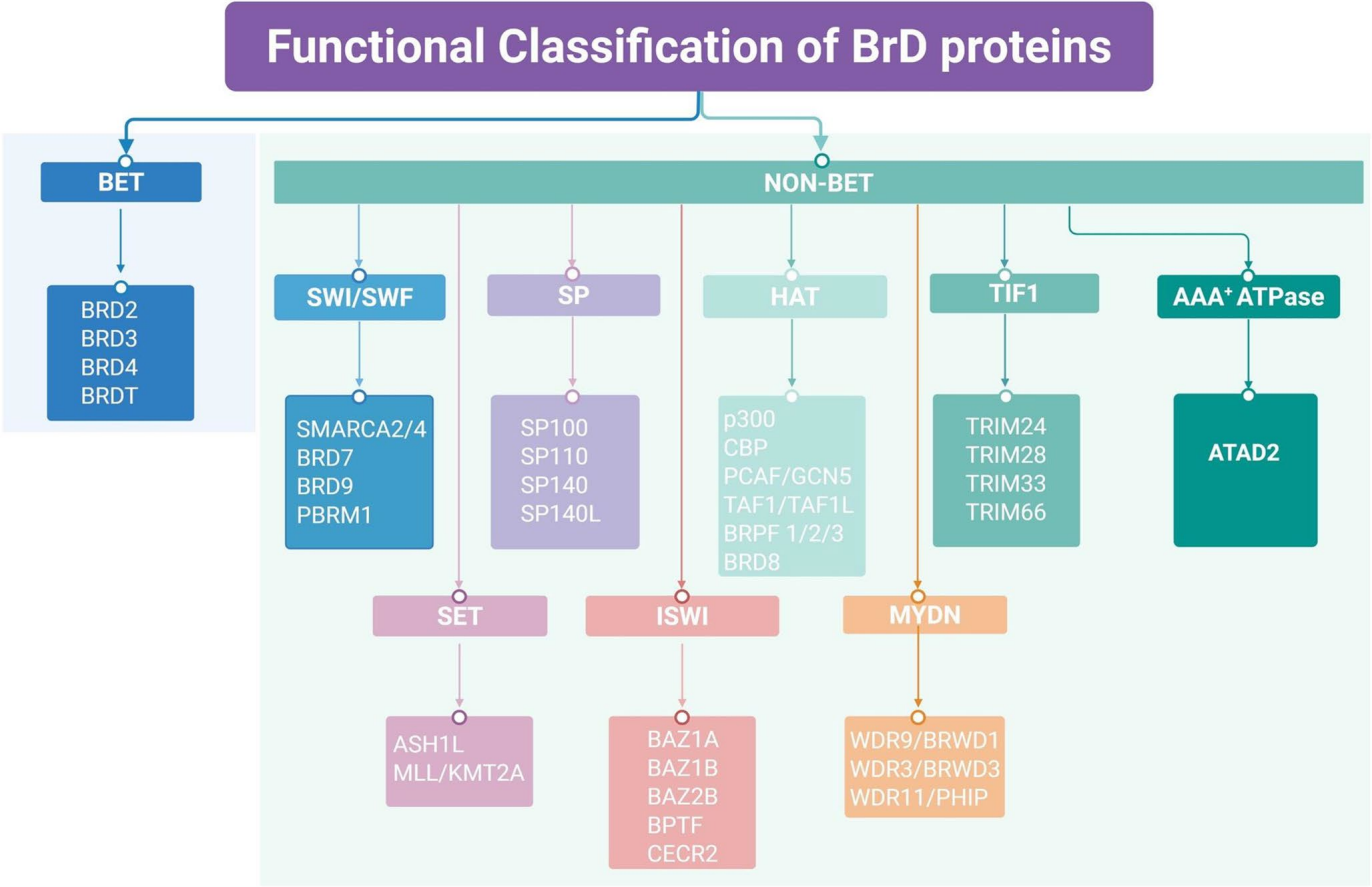
Functional classification of bromodomain (BrD) proteins. The BrD-containing proteins are categorized into two primary functional groups: BET and non-BET families. The BET family is characterized by tandem BrDs and an ET domain that facilitates transcriptional activation. In contrast, non-BET proteins are further divided accordingly to their predominant biological roles, including chromatin remodeling, histone acetylation, and other regulatory functions. This classification underscores the functional diversity of BrD proteins in regulating gene expression and chromatin-associated processes

**Fig. 3:**
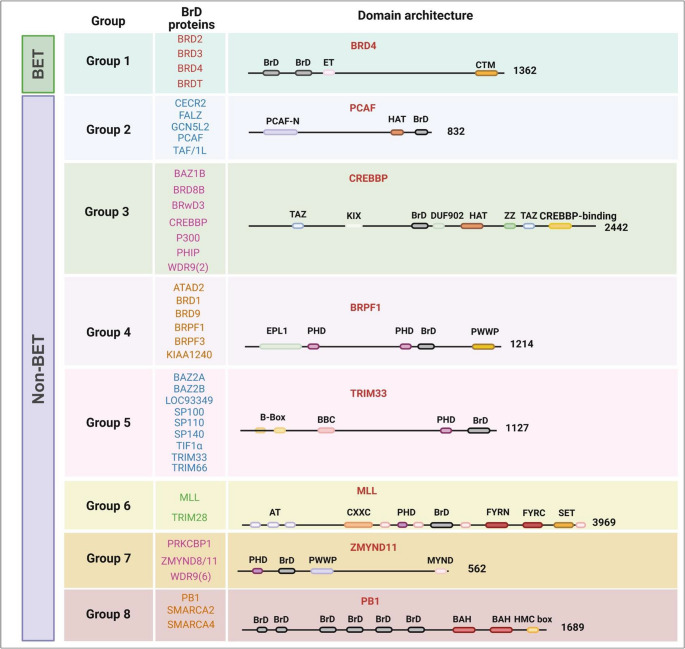
Domain architecture of bromodomain (BrD) family proteins. Representative BrD-containing proteins are shown. Numbers indicate the amino acid length of a BrD protein in each group. Domain abbreviations: PCAF, p300/CBP-associated factor; PCAF-N, PCAF N-terminal domain; HAT, histone acetyltransferase domain; BRD4, BrD-containing protein 4; ET, extra-terminal domain; CTM, carboxyl-terminal motif; CBP, CREB-binding protein; KIX, CREB-interacting kinase-inducible domain interacting domain; ZZ, ZZ-type zinc finger domain; TAZ, transcriptional adaptor zinc finger domain; PHD, plant homeodomain; BRPF1, BrD- and PHD finger-containing protein; PWWP, Pro-Trp-Trp-Pro domain; TRIM33, tripartite motif-containing 33; BB1/BB2, B-box domains 1 and 2; MLL, mixed lineage leukemia; AT-hook, DNA-binding domain; CxxC, zinc-finger motif (DNA binding, CpG interaction); FYRN/FYRC, Phe/Tyr-rich domains (mediate N–C terminal interactions); SET, Su(var)3–9, enhancer-of-zeste, trithorax (histone methyltransferase catalytic domain); ZMYND11, zinc finger MYND-type containing 11; MYND, Myeloid, Nervy, and DEAF-1 domain; PB1 or PBRM1, polybromo 1; BAH, bromo-adjacent homology domains; HMC, high-mobility group domain. Created with BioRender (https://app.biorender.com/)

The human genome encodes 61 BrD modules distributed across 46 proteins, each containing one to six BrDs [14]. Beyond their well-established roles in gene expression, BrD proteins exhibit functional diversity through their involvement in chromatin-associated mechanisms [20]. Based on sequence and structural similarities, these proteins are categorized into eight subfamilies [13, 14]. A detailed overview of their functional classification is illustrated in Fig. 2, while Fig. 3 depicts the domain architecture-based classification of BrD proteins. The functional attributes and classification of BrD proteins are summarized in Table 1.

**Table 1:** Functional classification of BrD proteins

BrD Family	Name of the protein	Classifications	No. of BrDs	Functions	References
Ia	p300/CBP	HAT-containing transcriptional co-activators	1	Histone acetyltransferase	[19]
	PCAF		1		[17]
	GCN5L2		1	Histone acetyltransferase, Transcriptional activator	[21, 22]
	TAF1		2	Transcription initiation	[23]
	TAF1L		2		[24]
Ib	BRPF1	BrD and PHD finger (BRPF) proteins	1	Transcriptional activator	[25]
	BRPF2		1	Transcriptional activator	[26]
	BRPF3		1	Component of MOZ/MORF H3 acetyltransferase complex	[27]
	BRD8		1	Transcriptional regulator	[28]
II	ASH1L	SET domain containing histone lysine methyltransferases	1	Histone Methyltransferase	[29]
	MLL		1	Histone Methyltransferase	[30]
IIIa	SMARCA2	SWI/SNF family of chromatin remodeling complexes	1	Chromatin remodelers and Splicing regulator	[31]
	SMARCA4		1	Chromatin remodelers	[32]
	BRD7		1	Transcriptional regulator and Tumor suppressor	[33]
	BRD9		1	Transcriptional regulator	[27]
	PBRM1		6	Chromatin remodelers	[34]
IIIb	BAZ1A/1B	ISWI family of chromatin remodeling complexes	1	Chromatin remodelers and Transcriptional regulator	[35]
	BAZ2A		1	Transcriptional repressor	[36]
	BAZ2B		1	Unknown	[37]
	BPTF		1	Chromatin remodelers, Transcription regulator	[38]
	CECR2		1	Chromatin remodelers	[39]
IV	ATAD2	AAA domain-containing ATPase family proteins	1	Transcriptional regulators	[40]
	ATAD2B		1	histone-binding factor	[41]
V	BRD2	BET family proteins	2	Transcriptional regulator	[42]
	BRD3		2	Transcriptional regulator	[42]
	BRD4		2	Transcriptional regulator	[43]
	BRDT		2	Chromatin remodelers	[44]
VI	TIF1-α (TRIM24)	Transcriptional Intermediary Factor 1 (TIF1)	1	Ubiquitin E3 ligase, Transcriptional regulator	[45]
	TIF1-β (TRIM28/KAP1)		1	Sumo E3 ligase, Transcriptional regulator	[46]
	TIF1-γ (TRIM33)		1	Ubiquitin E3 ligase, Control of Transcription elongation	[47]
	TIF1-δ (TRIM66)		1	Transcriptional repressor	[48]
VII	SP100	Speckled Protein (SP) family	1	Transcriptional regulator	[49]
	SP110		1	Transcriptional regulator	[50]
	SP140		1	Transcriptional regulator	[51]
	SP140L		1	Unknown	
VIII	ZMYND8	Myeloid, Nervy, and DEAF-1 (MYDN) proteins	1	Transcriptional regulator	[52]
	ZMYDN11		1	Transcriptional repressor	[53]
IX	WDR9 (BRWD1)	Myeloid, Nervy and DEAF-1 (MYDN) proteins	2	Chromatin remodelers	[54]
	WDR3 (BRWD3)		2	Chromatin remodelers	[55]
	WDR11 (PHIP)		2	Unknown	

### HAT-Containing Transcriptional Cofactors

HATs catalyze the acetylation of lysine residues on histone and transcription factors, a process facilitated by Group Ia BrD proteins. These proteins enhance HAT activity and contribute to chromatin decondensation and transcriptional activation. HATs often function within multiprotein complexes that include the basal transcriptional machinery (e.g., TFIIB, TBP, Pol II), specific transcription factors, acetyltransferases, and other coactivator complexes (like ARC) [56]. Prominent coactivators containing BrDs include CREB-binding protein (p300/CBP), PCAF, GCN5L2, and TATA-binding protein-associated factors (TAF1). These proteins not only promote the recruitment of HATs to acetylated chromatin but also aid in the propagation of acetylation marks, ensuring coordinated transcriptional activities [57].

#### p300/CBP

The CBP (also known as KAT3A) and p300 (EP300, also known as KAT3B) are paralogous KATs with multiple functional domains that serve as transcriptional co-activators [58]. The BrD, HAT, and plant homeodomain (PHD) together form the catalytic core. The BrD recognizes Kac residues and facilitates histone H3 and H4 acetylation, whereas the PHD in p300/CBP is structurally disrupted by an adjacent RING domain and is unlikely to function as a canonical H3K4me3 reader [13, 59]. Despite extensive study, the precise roles of the BrD and PHD in p300/CBP function remain controversial. Some studies report that BrD inhibition or deletion reduces chromatin association and global histone acetylation, indicating an essential scaffolding or allosteric role [60–62]. Conversely, other reports find minimal or context-dependent effects on HAT activity, implying compensation by other domains or cofactors [63, 64]. Similarly, several structural and biochemical studies indicate that the PHD-containing CH2 region, interrupted by a RING domain, regulates the HAT domain through intramolecular interactions [59, 65]. While some reports suggest the PHD contributes to catalytic integrity, others show that the adjacent PHD/RING region can autoinhibit HAT activity, such that its removal has little or even an enhancing effect [66, 67]. These inconsistencies may reflect differences in experimental systems, chromatin context, or the presence of compensatory protein interactions.

Given the complex regulation of these domains, mutations affecting them frequently result in pathological consequences [57]. Notably, driver mutations in cancers are frequently localized to the catalytic, BrD, or PHD regions. For example, the D1399N/Y substitution in p300 disrupts its essential autoacetylation functions, compromising its activity and contributing to oncogenesis [56, 58, 68]. Additionally, the BrD of p300/CBP plays a key role in regulating myeloid-derived suppressor cells (MDSCs) by controlling H3K27 acetylation, promoting tumor growth [56, 58, 68]. Inhibiting the BrD enhances MDSC inflammation and suppresses tumor growth. Together, these findings highlight p300/CBP as critical chromatin regulators whose structural integrity and enzymatic functions are central to both normal transcriptional control and cancer progression.

#### PCAF/GCN5

GCN5 (also known as KAT2A) and its paralog PCAF (also known as KAT2B) share significant sequence similarity, particularly in the HAT and BrD regions [13]. The PCAF contains an N-terminal E3 ubiquitin ligase domain that binds to p300/CBP, while GCN5 features an N-terminal domain for nucleosome recognition [14]. Both these proteins participate in multi-subunit complexes – ATAC and SAGA complexes – regulating histone acetylation at key sites [69]. While both acetylate histones, PCAF mainly targets H3 and H4, focusing on H3K14, H3K9, and H4K8, whereas GCN5 acetylates H2a, H2b, H3, and H4, with a preference for H3 and H4. GCN5 and PCAF are implicated in cancer through their roles in maintaining genomic integrity [15, 69, 70].

#### TAF1/TAF1L

Several TAFs are primarily located in lobes A and C of TFIID and are vital for transcription regulation [71]. A subset of TAFs is also present in SAGA complexes, further contributing to transcriptional control [71]. TAF1, the largest subunit of the TFIID complex, serves as a scaffold for other TAFs and the TATA-binding protein, facilitating preinitiation complex formation [72]. TAF1 and its homolog TAF1L are multidomain proteins featuring two kinase domains, a HAT domain, and two BrDs – one N-terminal and one C-terminal. The HAT domain in these proteins differ from that of other HAT family members and requires TAF7 for its function [13, 57]. The N-terminal and C-terminal BrDs of TAF1 and TAF1L share high sequence identity (>97%), whereas the BrDs within each protein (N-terminal vs. C-terminal) display lower identity (37–39%). Notably, this internal divergence includes a leucine-to-tyrosine substitution at the gatekeeper residue, which alters the architecture of the binding pocket and affects access to the hydrophobic WPF shelf [73].

TAF1 has diverse roles, interacting with MYC and p53 to regulate gene transcription and cell cycle transitions, and extending its acetyltransferase and ubiquitination activities to histones and transcription factors [57]. TAF1L, which replaces TAF1 in male germ cells during meiosis, binds to the TATA-binding protein and substitutes for TAF1 in temperature-sensitive hamster cell lines [13, 14].

#### BRPF1/2/3 and BRD8

BRPF1/2/3 and BRD8 belong to the BrD and PHD finger (BRPF) protein family, act as accessory subunits of the conserved eukaryotic MYST family HATs (MOZ/MORF, MOF, TIP60, and HBO1), involved in chromatin remodeling [13, 57]. BRPF proteins contain a tandem PHD domain separated by a mononuclear zinc knuckle (collectively, PZP domain). The PHD fingers, characterized by a conserved C3HC4 zinc finger motif, are commonly found in nuclear proteins that regulate transcription and chromatin structure [14]. BRPF1/2/3 proteins also have C-terminal BrDs, which share 56.3% sequence identity and 91.5% sequence similarity [13, 14], and a chromo/Tudor-related PWWP domain. The BrD of BRPF1 recognizes acetylated histone marks, particularly H2AK5ac, H4K12ac, and H3K14ac, while its PWWP domain binds to H3K36me3, facilitating chromatin targeting [57]. BRPF1 forms tetrameric complexes with one of the three HATs: MOZ (KAT6A), MORF (KAT6B), or HBO1 (KAT7), along with the accessory proteins ING5 or its paralog ING4, and MEAF6 [74]. Acetylation of chromatin likely enhances the affinity of the MOZ complex, as the BrDs recognize newly acetylated marks, creating a positive feedback loop. This promotes further recruitment of MOZ, increasing local acetylation and upregulating gene expression [75].

BRPF2 (also known as BRD1) and BRPF3 associate with the HBO1 HAT complex, promoting acetylation of H4 at K5, K8, K12, and H3 at K14, in cooperation with ING4 and hEAF6 subunits [76–78]. HBO1 functions primarily as an H4-specific acetyltransferase, essential for transcription and DNA replication [77]. In BRPF2, the first PHD finger (PHD1) recognizes the unmodified H3 tail, while the second (PHD2) binds with DNA in a non-specific manner [13, 57]. The role of the PZP domain in BRPF3 is still unknown. The BrD of BRPF2 preferentially binds Kac on histones, specifically H4K5ac, H4K8ac, and H4K5acK12ac [79]. The PWWP domain in BRPF1/2/3 binds H3K36me3, facilitating BRPF1’s association with condensed chromatin [74, 76, 78]. Although the BRPF3-HBO1 complex has recently been shown to acetylate H3K14 [77], the mechanism by which BRPF3 recruits HBO1 for specific transcriptional programs is not yet understood. Additionally, no histone ligand has been identified for the BRPF3 BrD to date. Moreover, BRD8 has been identified as a non-catalytic component of the conserved nucleosome acetyltransferase of H4 (NuA4) complex, a multisubunit HAT that acetylates the N-terminal tails of H4 and H2A [57].

### SET Domain Containing Histone Lysine Methyltransferases ASH1L and MLL

Absent, small, or homeotic-like 1 (ASH1L) and Mixed-lineage leukemia 1 (MLL, also known as KMT2A) are histone methyltransferases that contain aBrDs lacking the conserved asparagine residue required for canonical Kac recognition [13, 14]. Both proteins also harbor PHD and SET (Su(var)3–9, enhancer of zeste, and trithorax) domains responsible for methyltransferase activity [57]. In ASH1L, the aBrD is paired with a PHD finger and a BAH (Bromo-adjacent homology) domain, whereas in MLL, it is linked to a PHD finger and a FYRN (phenylalanine- and tyrosine-rich N-terminal) domain. The specific functions of these aBrD-PHD modules and associated domains remain unclear, requiring further investigation [80]. ASH1L forms the AMC (ASH1L-MRG15-CAF1) complex with MRG15 (a transcription factor) and CAF1 (a histone binding protein), which promotes histone lysine methyltransferases activity by destabilizing autoinhibitory loop [80]. In addition, ASH1L has been implicated in tumor progression across multiple cancer types and plays a key role in acute leukemia, particularly in cases involving MLL1 translocations, which are associated with poor prognosis [57].

In mammals, the MLL family proteins consist of six members, including MLL1-4 (KMT2A-D), SETD1A (KMT2F), and SETD1B (KMT2G) [57]. The histone lysine methyltransferases activity of these proteins is mediated by four conserved core subunits: WD-repeat containing protein5 (WDR5), retinoblastoma-binding protein 5 (RBBP5), ASH2L, and dumpy-30 (DPY30) [57]. Among these MLL proteins, MLL1 is crucial for transcriptional activation and plays a key role in various cancers [14, 57]. It maintains the H3K36me2 mark, aiding recruitment of the MLL1 complex to target genes, particularly in leukemogenesis [81]. Despite its importance and therapeutic relevance, no effective small-molecule inhibitors targeting MLL1 have been identified to date.

#### BrD Encoding Members of the SWI/SNF Family of Chromatin Remodeling Complexes (SMARCA2/4, BRD7, BRD9, and PBRM1)

The SWI/SNF chromatin remodeling complexes are composed of at least 29 distinct subunits and are essential for regulating cell growth and differentiation [82]. These complexes are categorized into three major subtypes – BAF, PBAF, and the recently discovered GBAF (also known as ncBAF) – each defined by specific subunit compositions. Core components such as SMARCA2/4 and SMARCC1 are shared across all subtypes, emphasizing their essential role in maintaining chromatin dynamics and cellular function.

SMARCA2 (BRM) and SMARCA4 (BRG1) are the core ATPase subunits of the SWI/SNF complex, driving chromatin remodeling through ATP hydrolysis and playing essential roles in transcription regulation [82, 83]. Both function as tumor suppressors, with SMARCA4 frequently mutated and SMARCA2 often silenced in a variety of cancers. Among subtype-specific subunits, BRD7 in the PBAF complex also acts as a tumor suppressor, and its downregulation correlates with poor prognosis in many cancer types. In contrast, BRD9, part of the ncBAF complex, promotes cancer progression, and its inhibition has been shown to suppress proliferation in certain cancers [82, 83]. PBRM1, another PBAF subunit, is frequently mutated in kidney cancer, where its loss, particularly alongside VHL (Von Hippel-Lindau) inactivation, contributes to tumorigenesis [82, 83]. The BrD module present in subunits such as BRD7 and BRD9 mediates the recognition of Kac residues on histone tails, facilitating recruitment of the SWI/SNF complex to open chromatin regions. Notably, pharmacological inhibition of the BRD9 BrD disrupts the occupancy of BRD9 and its associated ATPase SMARCA4 at lineage-specific chromatin loci, impairing differentiation programs such as melanocyte pigmentation [84, 85]. Conversely, in BRD9-containing SWI/SNF subcomplexes from pediatric malignant rhabdoid tumors, the BrD is not required for assembly; instead, the DUF3512 domain mediates subunit interactions [84]. Collectively, these findings underscore the critical role of SWI/SNF complex subunits in cancer development and progression.

#### BrD Encoding Members of the Imitation Switch Family of Chromatin Remodeling Complexes (BAZ1A, BAZ1B, BAZ2A, BAZ2B, BPTF and CECR2)

Imitation switch (ISWI) complexes regulate chromatin structure by facilitating pre-nucleosome maturation and maintaining proper nucleosome spacing, which is crucial for transcriptional regulation and the DNA damage response. ISWI family members, characterized by SWI2/SNF2 ATPase and HAND-SANT-SLIDE modules, mediate chromatin remodeling and nucleosome sliding [13, 57]. In mammals, ISWI complexes consist of a single ATPase subunit (SMARCA1 or SMARCA5) along with various noncatalytic accessory proteins that confer functional specificity [13, 14, 57].

BAZ1A forms ACF (ATP-utilizing chromatin assembly and remodeling factor) and CHRAC (chromatin accessibility complex) complexes to enhance nucleosome sliding during DNA replication and transcription, with its disruption potentially leading to tumor cell senescence via SMAD3 upregulation [86]. BAZ1B, a component of the WICH complex, is overexpressed in multiple carcinomas. It activates CYP19A1 in breast cancer and promotes tumor aggressiveness in lung cancer through the PI3K/Akt and IL-6/STAT3 pathways [86]. BAZ2A, a member of the nucleolar remodeling complex (NORC), enhances epithelial–mesenchymal transition (EMT) in hepatocellular carcinoma (HCC) and is associated with rDNA instability in various cancers, including prostate cancer and chronic lymphocytic leukemia (CLL) [86]. In contrast, BAZ2B is downregulated in kidney chromophobe (KICH), lung squamous cell carcinoma (LUSC), and breast cancer and frequently mutated in melanoma and colorectal adenocarcinoma (COAD) [86]. In this family, the BrD is functionally critical. For instance, the BrD of BAZ2A specifically binds the acetylated histone mark H3K14ac at inactive enhancer regions, thereby enabling recruitment of the BAZ2A-containing complex to chromatin and supporting a cancer stem-like state in prostate cancer [57]. Pharmacological inhibition or degradation of the BAZ2A BrD disrupts this interaction, leading to loss of stem-cell features and impaired tumor initiation in prostate organoid models [87–89]. BPTF, critical for chromatin remodeling and c-MYC recruitment, is upregulated in stomach adenocarcinoma (STAD), esophageal carcinoma (ESCA), liver hepatocellular carcinoma (LIHC), and lung adenocarcinoma (LUAD), while downregulated in KICH and prostate adenocarcinoma (PRAD). It also exhibited a high mutation frequency in angiosarcoma and COAD, and its knockdown has been shown to impair tumor development [86]. CECR2 has emerging roles in breast cancer metastasis and macrophage-mediated immune suppression [76]. The involvement of BrD modules in these ISWI-associated subunits highlights the significance of acetyl-lysine recognition in directing nucleosome remodelers to specific chromatin sites.

### AAA Domain-Containing ATPase Family Proteins

AAA + ATPases are hexameric enzymes that mediate ATP-dependent hydrolysis, playing key roles in diverse cellular processes [90]. ATAD2, a type II AAA + ATPase, regulates chromatin dynamics by modulating histone-DNA interactions. It contains both an AAA + domain and a BrD [90]. ATAD2 and its paralog ATAD2B share high sequence similarity but differ in histone ligand specificity, with ATAD2 preferentially binding H4K5ac, while ATAD2B favors H4K12ac. Both proteins recognize the diacetylated H4K5acK12ac mark, although their binding preferences suggest distinct functions in chromatin regulation [90]. ATAD2 is frequently overexpressed in various cancers, where it promotes tumor growth and is associated with poor prognosis [90]. It interacts with transcription factors such as ERα, AR, E2Fs, and c-Myc to drive proliferation and inhibit apoptosis. In certain cancers, ATAD2 enhances proliferation via the PI3K/AKT and Rb-E2F-cMyc signaling pathways and regulates glucose metabolism [90]. Silencing ATAD2 reduces tumor growth, highlighting its potential as a therapeutic target. In contrast, the role of ATAD2B in cancer remains largely undefined.

### BET Family Proteins (BRD2, BRD3, BRD4, and BRDT)

The BET protein family comprises four members – BRD2, BRD3, BRD4, and BRDT – primarily involved in transcriptional activation and RNA polymerase II regulation [91, 92]. BET proteins are defined by two conserved N-terminal BrDs (BD1 and BD2) and a unique C-terminal extraterminal (ET) domain [57, 92]. BRD2 and BRD3 regulate the cell cycle by influencing E2F proteins and cyclin D1, while BRD4 aids transcription elongation through its interaction with the p-TEFb complex [92]. Additionally, BRD2 and BRD3 contribute to nucleosome assembly during DNA replication, while BRDT and BRD4 facilitate chromatin remodeling at hyperacetylated nucleosomes [91, 92]. BRD2 and BRD4 remain associated with chromatin throughout the cell cycle, supporting the maintenance of epigenetic information. BRDT, expressed during meiosis, represses H1t expression by interacting with regulatory proteins [91, 92]. Together, these findings establish the BET family as key chromatin readers that coordinate transcriptional regulation to sustain proper cell cycle progression and gene expression fidelity.

### TIF1 (transcriptional Intermediary Factor 1)

TIF1 proteins belong to the tripartite motif (TRIM) superfamily of E3 ubiquitin ligases and comprise four subtypes: TIF1-α (TRIM24), TIF1-β (TRIM28/KAP1), TIF1-γ (TRIM33), and TIF1-δ (TRIM66). All four fall under the C-VI subfamily and share a conserved domain architecture consisting of an N-terminal TRIM domain, a central TIF1 signature sequence (TSS) domain, and a C-terminal PHD-BrD module [93]. The really interesting new gene domain (RING) domain within the TRIM region confers E3 ligase activity, enabling substrate ubiquitination via the ubiquitin-proteasome pathway [93]. The B-box and coiled-coil domains facilitate protein-protein interactions, supporting complex assembly and functional specificity.

Members of the TIF1 family are often dysregulated in various cancers, affecting prognosis and representing potential therapeutic targets. TRIM24 is overexpressed in several cancer types, correlating with aggressive tumor phenotypes, but may act as a tumor suppressor in murine HCC [93]. TRIM28 is highly expressed in ovarian, breast, and gastric cancers and is linked to poor prognosis and aggressive clinical features. While some studies in early-stage lung cancer suggest a correlation with better survival, elevated TRIM28 levels more broadly correlate with enhanced cancer stemness and worse outcomes across solid tumors [93]. TRIM33 functions as a tumor suppressor in HCC, and its low expression is associated with genomic instability in breast and pancreatic cancers, as well as in multiple myeloma and chronic myelomonocytic leukemia [93]. TRIM66 is overexpressed in osteosarcoma and non-small cell lung carcinoma (NSCLC), where it is linked to metastasis and reduced survival; knockdown studies support its oncogenic role in various malignancies [93].

### Speckled Protein (SP) Family

The SP family consists of SP100, SP110, SP140, and SP140-like protein (SP140L), all of which contain a nuclear localization signal and several conserved functional domains, including the rare SAND domain for DNA interaction, along with a PHD and a BrD [94]. Additionally, these proteins feature a caspase activation and recruitment domain (CARD) that promotes multimerization. SP100 and SP110 are broadly expressed in both immune and non-immune cells, whereas SP140 expression is restricted to immune cells [13, 14]. Members of the SP family are components of promyelocytic leukemia nuclear bodies (PML-NBs), which are involved in chromatin organization and gene expression, particularly under cellular stress conditions like virus infection or DNA damage [94].

Mechanistic studies show that the PHD-BrD cassette in SP100 preferentially recognizes unmethylated H3K4, rather than H3K4me3, indicating that the BrD primarily plays a structural role in stabilizing the PHD fold rather than mediating canonical acetyl-lysine recognition [57]. SP100 has 11 isoforms and undergoes various post-translational modifications that modulate its functions, while SP140 activity is specifically enhanced through SUMOylation, which dynamically regulates its histone binding and chromatin association through the BrD module [13, 94]. Although SP family proteins play established roles in innate immunity signaling pathways [95], their contribution to tumorigenesis remains poorly understood.

### Myeloid, Nervy and DEAF-1 (MYDN) Proteins

The WDR family includes WDR9 (BRWD1), WDR3 (BRWD3), and WDR11 (PHIP), all characterized by a β-propellor-shaped WD repeat domain [96, 97]. Unlike BET family proteins, these BRWD proteins contain tandem BrDs, comprising one canonical Kac-binding domain and another of unknown function. WDR9 regulates over 7,000 genes essential for B cell differentiation and proliferation and acts as a transcriptional regulator through its interaction with BRG1, a core component of the SWI/SNF chromatin remodeling complex [13, 57]. BRWD3 is upregulated in the plasma of breast cancer patients, indicating its potential utility as a serological biomarker [14, 57]. PHIP has been implicated in the progression of multiple tumor types, including breast cancer, lung cancer, and melanoma; its downregulation reduces AKT phosphorylation and cyclin D1 expression levels [97]. Increased PHIP expression correlates with poorer survival outcomes in HER2 + breast cancer [97]. Overall, the WDR family of BrD-containing proteins remains an underexplored group with emerging roles in cancer and developmental disorders.

### BrDs: Key Players in Stem Cell Identity and Differentiation

Stem cells are characterized by their ability to self-renew and differentiate into various specialized cell types. They originate either from embryos as pluripotent stem cells, such as embryonic stem cells (ESCs), which can differentiate into multiple cell types across all three germ cells, or from adult tissues as multipotent stem cells, which differentiate in a lineage-restricted manner. Additionally, somatic cells can be reprogrammed into induced pluripotent stem cells (iPSCs), which are like ESCs and hold the potential for regenerative medicine [98, 99].

### BrD in Pluripotent Stem Cell

The unique characteristics of pluripotent stem cells are defined by their distinctive epigenomic and transcriptomic profiles. A critical factor in determining pluripotent stem cell identity is the presence of a transcriptionally permissive open chromatin state, maintained by a robust network comprised of chromatin remodeling complexes and pluripotency transcription factors, facilitating transcriptional activation [100–102]. The BrD family of proteins facilitates the opening of chromatin by recruiting chromatin remodelers, which promotes the expression of pluripotency genes [103]. The core pluripotency transcription factors like OCT4, SOX2, NANOG, KLF4, and ESRRB coordinate clusters of enhancers that are in close genomic proximity at multiple pluripotency genes, governing the transcriptional program responsible for maintaining the undifferentiated state of ESCs [104, 105]. These clusters of enhancers associated with key pluripotency genes exhibit high levels of histone acetylation marks and have a high density of coactivator proteins (such as the mediator complex) and master transcription factors binding sites [104]. The BrD-containing coactivators, p300 and CBP, regulate transcription through their HAT activity, which acetylates all four histones, including H3K56, a modification linked to ESC master transcription factors [106]. They also interact with NANOG and facilitate the formation of long-range loops crucial for gene activation. These functions of the coactivator are essential for maintaining the pluripotency of ESCs. However, their association factor PCAF has no impact on pluripotency [69].

PCAF may exhibit functional redundancy with GCN5 in ESCs, as the loss of *Gcn5* in mouse embryos does not disrupt blastocyst development or the maintenance of mouse ESCs [107, 108]. However, it plays a crucial role in regulating MYC-dependent networks in stem cells by preferentially interacting with MYC and E2F1 motifs, thereby influencing the expression of their target genes [107]. Additionally, GCN5 cooperates with N-MYC to regulate genes associated with the RAS and WNT signaling pathways by regulating H3K9ac levels [109]. Intriguingly, histone acetylation recruits BrD protein ATAD2 to chromatin, increasing accessibility and facilitating histone dynamics to maintain open chromatin and ensure the proper function of highly expressed genes in ESCs [110]. ATAD2 is non-essential for proliferation in exponentially growing ESCs, but it is crucial for maintaining gene expression programs that govern proliferation during differentiation. Its depletion triggers compensatory upregulation of helicase-related genes and downregulation of E2F-regulated proliferation genes.

Although histone acetylation maintains the open chromatin state necessary for pluripotency, BrD-containing protein BRPF2 specifically regulates global H3K14 acetylation [111]. Loss of *Brpf2* in mouse ESCs impairs differentiation, resulting in abnormal embryoid formation, downregulation of differentiation-associated genes, and sustained alkaline phosphatase activity. Consequently, BRPF1 is essential for mouse embryogenesis and gene regulation [112], whereas BRPF3 was initially considered nonessential for development, later revealed its role in ESC differentiation and cell cycle progression through stabilizing *Myst2* acetyltransferase [113, 114]. Proper differentiation of ESCs also relies on BPTF, a component of the NURF complex, with its loss resulting in embryonic lethality [115]. Meanwhile, another BrD protein, MLL1, along with SET1, BACH, and NANOG, regulates the H3K4 tri-methylation state, facilitating enhancer-promoter activity and ensuring the expression of stemness-related genes to support ESC pluripotency [116].

In ESCs, ~ 30% of the genes initiate transcription but fail to undergo transcriptional elongation as RNA polymerase II (RNA pol II) stalled at their promoters, including pluripotency genes [117]. Interestingly, BRD4 reads and binds to the Kac residues on histones at these clusters of enhancers via its BrD and recruits the mediator complex along with Cyclin-dependent kinase 9 (CDK9), a pause release factor phosphorylating the C-terminal domain (CTD) of RNA pol II, to promote transcriptional elongation in ESCs [104, 105, 118–120]. This intricate regulatory mechanism ensures the maintenance of ESC pluripotency and regulates cell fate decisions. Besides binding to acetylated histones, the chromatin reader BRD4 also interacts with histone acetyltransferases p300 and CBP to enhance their enzymatic activities, resulting in increased histone acetylation [103]. This BRD4-p300-mediated histone modification alters chromatin structure by recruiting ATP-dependent chromatin remodeler BRG1, thereby maintaining the expression and chromatin patterns of genes associated with pluripotency, such as *Nanog*, *Oct4*, *Sox2*, and long non-coding RNAs, *Tsix* and *Xite*, involved in X chromosome inactivation. BRD4 also plays a pivotal role in ESC self-renewal and fate determination by positively modulating the expression of stem cell-specific genes such as *OCT4* and *PRDM14* [119]. Moreover, protein-protein interaction analysis in mouse ESCs revealed that BRD4 interacts with the master pluripotency factor OCT4 and occupies the regulatory regions of numerous pluripotency-associated genes, including *Nanog*, *Oct4*, and so forth [121]. In mouse embryos and ESCs, BRD4 plays a crucial role in regulating *Nanog* expression [122]. In fact, BRD4 associates with BRG1 in the regulatory region of *Nanog*, promoting its expression by inhibiting repressive histone modification H3K27me3. Genome-wide mapping through chromatin immunoprecipitation-sequencing (ChIP-seq) revealed that in mouse ESCs, BRD4 and acetylated H4 co-occupies the upstream of core pluripotency genes such as *Nanog* and *Oct4* [123]. Upon differentiation, there is a simultaneous decrease in both BRD4 and global acetylated H4 levels, which signifies the importance of BRD4 in regulating pluripotency.

On the other hand, BRD4 inhibition decreases the recruitment of mediator complexes and CDK9 to the large clusters of regulatory elements in ESCs without affecting global acetylated H4 levels [119]. This leads to downregulating the cluster of enhancers-controlled pluripotency-associated genes, resulting in loss of pluripotency. In addition, chemical compound-mediated inhibition of the BET domain diminishes the expression of pluripotency genes, prompting rapid neuroectodermal lineage differentiation [119, 121]. Moreover, BET inhibitors induce the differentiation of mouse ESCs by drastically decreasing the expressions of the pluripotency factor *Nanog* and stemness marker *Lefty* [124]. Exceptionally, the expression of c-MYC, a gene known for its role in promoting proliferation and survival, remained unaffected following BET inhibition in both mouse and human ESCs despite being typically suppressed in cancer-related contexts by similar compound treatments [119, 124]. Knockdown or inhibition of BRD4 not only induces aberrant differentiation but also abolishes the self-renewal ability of pluripotent stem cells due to reduced *Nanog* levels [103]. During mesoderm lineage differentiation of ESCs, BRD4 modulates the chromatin by facilitating the accumulation of epigenetic marker H3K27Ac at the regulatory region of mesoderm master factor *Brachyury*, thereby promoting its expression.

Furthermore, dynamic switching between BRD4 and BRD2 regulates the fate of stem cells, with BRD4 facilitating stemness while BRD2 promotes Nodal signaling and mesendoderm development [125]. The downregulation of BRD4 in mouse ESCs led to the exit from pluripotency, and BET inhibition disrupts these processes, offering potential therapeutic applications. In the end, BRD4 plays a pivotal role in regulating pluripotency. However, in a subsequent study, a contrasting perspective was proposed that *Brd4* might not be necessary to maintain self-renewal and pluripotency in naïve ground state ESCs under certain specific conditions [126]. This scenario involves activating a network of pluripotency-specific transcription factors in conjunction with Tet1/2 DNA methylcytosine oxidases, leading to the constitutive decondensation of chromatin and recruitment of the mediator complex, independent of BRD4.

**Fig. 4:**
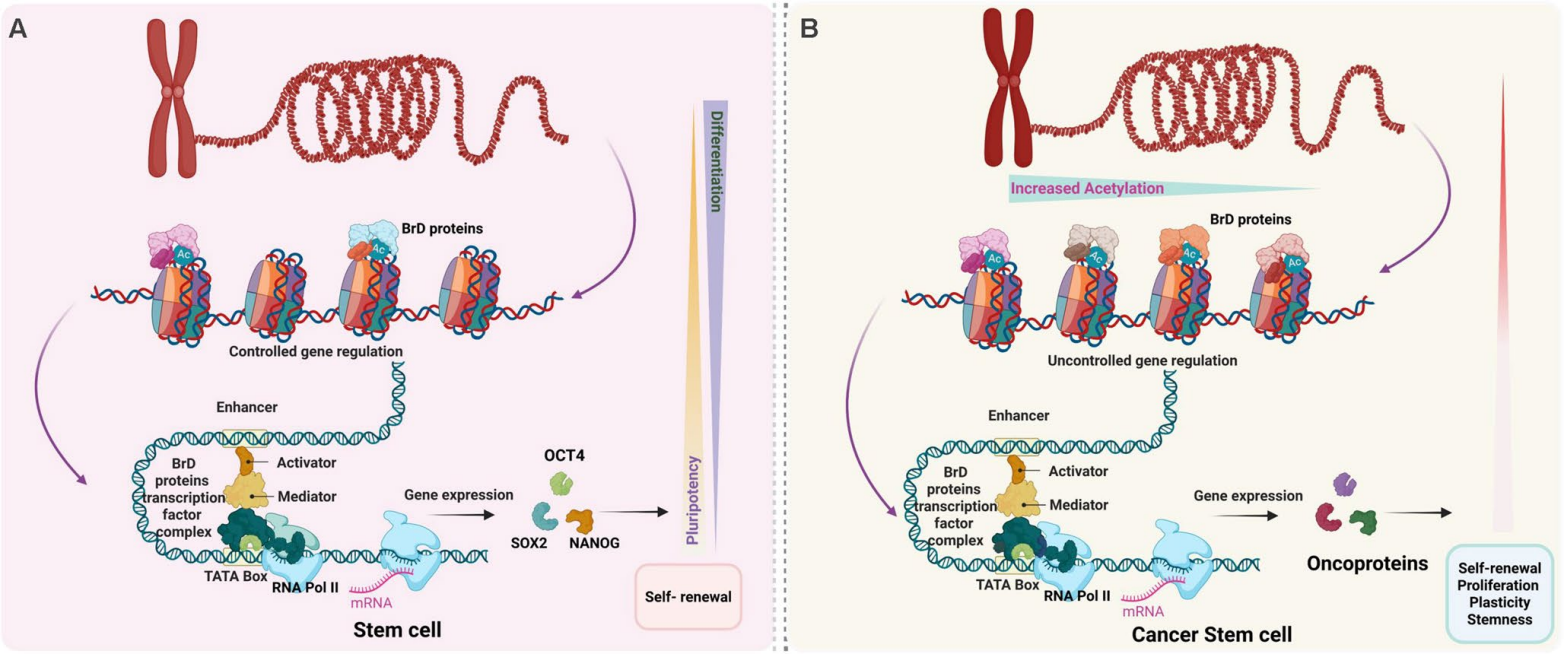
Bromodomain (BrD) proteins regulate stem cells (SCs) and cancer stem cells** (**CSCs).**A)** Schematic illustration of BrD protein-driven mechanisms in SCs. BrD protein complexes recognize acetylated lysine residues and regulate transcriptional processes. For instance, BRD9, BRD4 and/or p300 form complexes that act as backbone to recruit activators, mediators and transcription factors to promoter or enhancers regions, thereby controlling the expression of pluripotency genes such as *OCT4*,*SOX2*, and *NANOG.* This regulation, driven by acetylation of associated genes, maintains pluripotency and directs differentiation. **B) **Overview of BrD protein-mediated mechanisms in CSCs. In CSCs, aberrant acetylation leads to dysregulated BrD protein activity, resulting in uncontrolled transcription of oncogenes, enhanced proliferation, plasticity, stemness, and tumorigenic potential. Created using BioRender (https://app.biorender.com/)

Moreover, in ESCs, SMARCA4 is also essential for self-renewal, with its deletion leading to early embryonic lethality, whereas SMARCA2 knockout mice remain viable into adulthood [127]. Similarly, BRD7 plays a crucial role in regulating self-renewal by modulating gene expression profile, while PBRM1 loss results in embryonic lethality due to heart defects [33, 128]. The ncBAF complex supports naïve pluripotency in mouse ESCs, and its inhibition induces a primed epiblast-like state [129]. Additionally, BRD9 maintains somatic cell identity, and its suppression reduces chromatin barriers, facilitating reprogramming to pluripotency [130]. Equally, TRIM24 is a key component of the pluripotency network in mouse ESCs, where it collaborates with *Oct-3/4*, *Sox2*, and *Nanog* to activate cell cycle genes while suppressing developmental genes, thereby maintaining self-renewal [131]. Its upregulation enhances somatic cell reprogramming to iPSCs, emphasizing its role in establishing pluripotency [132]. Likewise, TRIM28 supports pluripotency by activating stemness markers and repressing differentiation genes, though its downregulation enhances reprogramming kinetics without sustaining stemness [133–135]. Conversely, TRIM33 primarily regulates ESC differentiation, as its loss impairs differentiation while maintaining self-renewal [136]. In parallel, ectopic expression of TAF1 promotes differentiation, marked by fewer alkaline phosphatase-positive colonies, increased differentiation marker expression, and differential regulation of genes, such as a decrease in OCT4 expression [137]. Collectively, in pluripotent stem cells, these studies highlighted the role of BrD-containing proteins in organizing chromatin structure, facilitating transcriptional regulation, and determining cell fate. Thus, BrD plays dual roles in shaping both the transcriptome and epigenome, a crucial aspect in pluripotent stem cell self-renewal and differentiation ability (Fig. 4A).

### BrD in Multipotent Stem Cell

#### Hematopoietic Stem Cells

Hematopoiesis is a complex and essential process of blood cell production and renewal. It relies on the subset of multipotent stem cells, hematopoietic stem cells (HSCs) – a precursor to all types of blood cells [138]. HSCs are tightly regulated by the intricate network of chromatin remodelers, transcription factors, and coactivators, which enable the activation and repression of transcription [139, 140]. Transcription coactivators p300/CBP are crucial for maintaining HSC, with p300 and mediator complex (MED12) interacting robustly on chromatin to maintain the active state of hematopoietic enhancers [141]. Depletion of MED12 destabilizes p300 binding at lineage-specific enhancers, leading to H3K27ac reduction, enhancer inactivation, and loss of HSC stemness markers such as ERG, ETV6, RUNX1, and TCF7. However, CBP and p300 play different roles in the regulation of HSCs, with CBP facilitating self-renewal and p300 being necessary for proper differentiation of HSCs, while both contribute to homeostasis of the hematopoietic microenvironment [142–145]. In this context, p300-MYB interaction regulates HSC proliferation and differentiation, and its disruption affects HSC homeostasis by regulating megakaryocytosis and thrombocytosis and reducing circulating thrombopoietin levels [143]. CBP, together with BRCA1, maintains normal hematopoiesis, and its haploinsufficiency disrupts the bone marrow microenvironment in mice, leading to myeloproliferative with increased splenic HSCs and a lethal systemic inflammatory disorder [144, 145]. Likewise, BRPF1 is vital for hematopoiesis, as its deletion in murine blood cells leads to early lethality due to acute bone marrow failure and aplastic anemia [146]. Loss of BRPF1 results in a drastic reduction in HSCs and progenitors with increased oxidative stress and apoptosis and downregulates key multipotency genes, such as *Slamf1*, *Hoxa9*, *Egr*,* Mecom*, *Hlf*, *Gata3*, and *Gfi1*.

GCN5 is essential for hematopoietic cell development, regulating myeloid and erythroid differentiation. It impairs granulocyte differentiation by acetylating CCAAT Enhancer Binding Protein Alpha (C/EBPα), interacts with ZBP-89 to control erythroid maturation, and shows increased expression during erythroid differentiation [147, 148]. Additionally, GCN5/PCAF influences monocyte and macrophage differentiation through the SAGA complex and modulates innate immune signaling by targeting TBK1, highlighting its broad impact on hematopoiesis and immune function [149, 150]. ASH1L is also crucial for maintaining the HSC quiescence and supporting long-term hematopoiesis, as its loss results in adult HSCs depletion, reduces cyclin-dependent kinase inhibitor 1b/c (*Cdkn1b/1c*) expression, and prevents bone marrow reconstitution after transplantation [151]. Although HSC function is impaired, *Ash1l-*deficient mice show increased niche availability, allowing efficient engraftment of transplanted wild-type HSCs. However, while *Ash1l* loss alone does not trigger overt hematopoietic failure, its combined deficiency with mixed-lineage leukemia 1 (*Mll*) causes rapid bone marrow collapse.

In HSCs, the BET protein BRD4 is essential for their generation, expansion, and functional maintenance, as its loss disrupts progenitor development and lymphoid differentiation while having a mild impact on macrophages [152]. Inhibiting or depleting BRD4 impairs T-cell development, reduces B-cell maturation, and decreases BCL2 expression, leading to increased apoptosis [153]. In fact, BRD4 inhibition expands the HSC pool, potentially by downregulating *Myb*, though this mechanism requires further investigation. Moreover, BRD4 disruption in ESCs impairs spontaneous differentiation and haematoendothelial potential, partly through c-MYC, a key regulator of HSC self-renewal, niche interactions, and hematopoiesis [154]. However, the inability of c-Myc overexpression to fully rescue hematopoietic defects in BRD4-deficient ESCs suggests that the role of BRD4 extends beyond c-Myc regulation [155]. This implies that BRD4 may regulate hematopoiesis through additional factors while itself being subject to epigenetic regulation during hematopoietic differentiation.

Interestingly, overexpression of BAZ2B activates essential gene-expression programs and induces a multipotent state in lineage-committed hematopoietic progenitors, enhancing their stemness, clonogenic potential, and engraftment efficiency [156]. In parallel, BPTF is essential for the maintenance and self-renewal of adult hematopoietic stem cells and mammary gland stem cells by driving a stemness gene regulatory network [157]. Together, these findings highlight the pivotal role of BrD-containing proteins in regulating stem cell identity, renewal, and functional competence within the hematopoietic system, demonstrating their essential contribution to the regulation of hematopoiesis.

#### Non-hematopoietic Stem Cells

Mesenchymal stem cells (MSCs), also referred to as multipotent stromal cells, mesenchymal stromal cells, or bone marrow stromal cells, are a population of non-hematopoietic, multipotent cells with fibroblast-like morphology [158]. They primarily reside within the bone marrow microenvironment, providing structural support and playing a crucial role in regulating hematopoietic stem and progenitor cell function [159]. Although MSCs were initially isolated from bone marrow, they have since been derived from a broad range of mesenchymal tissues, including the umbilical cord, adipose tissue, placenta, amniotic fluid, skeletal muscle, skin, liver, lungs, cervical tissue, dental pulp, synovium, periodontal ligaments, and dermis [159, 160]. Their high proliferative capacity, extensive differentiation potential, immunomodulatory properties, and absence of major ethical concerns make them highly suitable for cell-based therapies [161]. MSCs can differentiate into mesodermal lineages, such as osteoblasts, chondrocytes, and adipocytes, and demonstrate the ability to transdifferentiate into cell types of ectodermal and endodermal origin, highlighting their versatility in regenerative medicine [162]. Despite their therapeutic potential, a major challenge remains in developing accurate strategies to direct MSC differentiation toward specific cell fates [163]. Overcoming this challenge requires a deep understanding of the molecular and epigenetic mechanisms that control cell fate decisions and lineage specification.

BrD-containing proteins play a pivotal role in regulating MSC lineage specification by modulating chromatin structure and controlling gene expression programs [164–167]. CBP and p300 function as crucial transcriptional co-activators that regulate MSC lineage specification. These co-activators interact with SOX9 through its carboxyl-terminal activation domain in a cell type-dependent manner, promoting *COL2A1* expression, which encodes type II collagen – a hallmark of chondrogenesis – with ChIP studies confirming p300 occupancy at the *COL2A1* promoter [168]. Disruption of the CBP/SOX9 complex impairs *COL2A1* transcription and inhibits chondrogenic differentiation. p300 further facilitates chondrogenesis by enhancing histone acetylation levels at the *COL2A1* locus, thereby promoting transcriptional activation [164]. In adipogenesis, depletion of CBP or p300 decreases PPARγ-target gene expression and inhibits adipocyte differentiation [169]. Co-activators such as p300, GCN5, and PCAF are recruited to the PPARG2 and PRDM16 promoters, increasing histone acetylation to promote adipogenic gene expression, while their depletion disrupts this process [165, 166, 170]. Furthermore, the downregulation of HDAC1, HDAC2, and HDAC5 during adipogenic differentiation highlights the importance of dynamic histone acetylation in MSC lineage specification [167].

In the context of osteogenesis, p300 contributes by forming a transcription complex with RUNX2 and facilitating H3 acetylation at its promoter [171]. This recruitment is mediated by c-Jun, which guides p300 to the RUNX locus, leading to increased H3K27 acetylation and activation of osteogenic gene expression [172]. GCN5 plays an essential role in osteogenesis by acetylating regulatory regions of DKK1 and WNT pathway genes, thereby activating osteogenic transcriptional programs in periodontal ligament stem cells [173] and bone marrow-derived MSCs [174]. Loss of GCN5 impairs bone-forming capacity, contributing to conditions such as periodontitis and osteoporosis, while pharmacological interventions like aspirin restore its expression and osteogenic potential [173, 174]. PCAF also contributes to osteogenic differentiation by promoting H3K9 acetylation at BMP signaling gene loci, and its reduced expression – observed in aged and ovariectomized mice – is associated with impaired bone formation both in vitro and in vivo [175].

Following the role of co-activators in MSC differentiation, BRD4 – another chromatin-associated, BrD-containing regulatory protein – has been identified as a key modulator of WNT signaling and its downstream pathways in MSCs [176, 177]. WNT signaling is essential for orchestrating developmental processes and plays a crucial role in maintaining stem cell self-renewal and multipotency by establishing a supportive niche environment [176]. In umbilical cord-derived MSCs, pharmacological inhibition of BRD4 using BET inhibitors suppresses WNT signaling components, resulting in impaired self-renewal, reduced mitotic activity, disrupted intracellular signaling, and induced cell cycle arrest at G1-phase [177]. This proliferative defect is largely attributed to reduced expression of c-MYC – a central regulator of cell cycle progression and a direct transcriptional target of BRD4 – highlighting the crucial role of c-MYC in maintaining MSC proliferation. Interestingly, this contrasts with ESCs, where BRD4 inhibition does not significantly affect c-MYC levels, suggesting distinct, c-MYC-dependent regulatory mechanisms in MSCs [119, 124, 177]. Consistent with observations in ESCs, BET inhibition in MSCs also leads to substantial downregulation of core pluripotency factors such as OCT4, SOX2, and NANOG, reflecting a conserved role of BET proteins in maintaining stemness-associated transcriptional programs across stem cell types. Strengthening the link between c-MYC suppression and G1 arrest, BET inhibition with JQ1 also induces upregulation of the cyclin-dependent kinase inhibitor p21, thereby promoting G1-phase cell cycle arrest [178]. In contrast, JQ1 exposure in neuronal derivatives of MSCs leads to apoptosis rather than arrest, characterized by reduced expression of p21 and p53 and increased levels of the pro-apoptotic protein BAX [178]. This shift promotes mitochondrial cytochrome C release and subsequent activation of caspase-9, ultimately resulting in programmed cell death.

Moreover, expanding the role of BrD-containing proteins in MSC lineage commitment, ASH1L has been shown to promote osteogenic and chondrogenic differentiation while suppressing adipogenesis [179]. In osteoporotic bone, ASHIL expression is markedly reduced, correlating with decreased bone mass. Functional studies in C3H10T1/2 cells demonstrate that ASH1L exerts its effects through its SET domain, enhancing H3K4me3 enrichment at the promoters of key transcription factors involved in skeletal development. Collectively, these studies illustrate that BrD proteins exert stage-specific regulatory control over MSC fate by coordinating the expression of pluripotency-associated transcription factors, cell cycle mediators, and apoptotic pathways throughout differentiation.

### BrD in Cancer Stem Cells

CSCs possess characteristics of self-renewal, high proliferation, and the ability to initiate tumor growth [180]. The concept of CSCs emerged from two pivotal studies. The first, led by Dick and colleagues, demonstrated that a single cell could instigate tumor formation in mice, identifying specialized cancer cells with stem cell-like properties in AML. These cells, expressing CD34^+^/CD38^-^ markers, were successfully transplanted into mice. The second study, conducted by Clark et al., identified a small population of breast cancer cells expressing CD44^+^/CD24^-^ markers, highly tumorigenic compared to others, revealing the presence of stem cell-like cancer cells in solid tumors. Subsequently, CSCs were identified in brain, melanoma, and colon cancers, showing their heterogeneity and tumorigenic potential [180].

Core pluripotent transcription factors such as OCT4, SOX2, NANOG, KLF4, and MYC are frequently overexpressed in CSCs, contributing to self-renewal, tumorigenesis, and differentiation potential, and are often associated with poor clinical outcomes [181–183]. Among these, MYC plays a key role in tumor initiation and represents a potential therapeutic target [184]. Like normal stem cells, CSCs depend on key developmental signaling pathways, including Wnt, Notch, Hedgehog, NF-κB, and PI3K/PTEN, to maintain their stemness and plasticity [185, 186]. Beyond these pathways, epigenetic mechanisms such as DNA methylation, histone modifications, and the activity of non-coding RNAs also play a vital role in regulating CSC functions [187]. Post-translational histone modifications, particularly acetylation and methylation, modulate chromatin accessibility, aiding in the maintenance of CSCs and facilitating evasion of immune responses and drug resistance, further mirroring the complex regulatory systems observed in normal stem cells [188].

The BrD proteins, particularly those in the BET family, are well-established epigenetic regulators that control transcriptional programs associated with cancer progression and stem cell maintenance (Fig. 4B) [189]. BET proteins have been extensively studied for their role in sustaining cancer stem-like traits, including self-renewal, resistance to therapy, and tumor initiation. In contrast, the functional contribution of non-BET BrD proteins to cancer stemness is less well-defined. A recent study by Patrycja et al. analyzed 41 BrD family members and found that only 11 showed either positive or negative correlations with cancer stemness across diverse cancer cell types. Notably, the expression of BrD proteins varied significantly across different tumor lineages. Exceptions include five genes – *ATAD2*, *BAZ1A*, *KAT2A*, *SMARCA4*, and *TRIM28* – which were consistently upregulated, and two genes – *KAT2B* and *SMARCA2* – which were consistently downregulated, irrespective of tumor type [189].

Among the BET family, BRD4 has been widely characterized for its association with stem cells and stem cell-like properties in cancers [189, 190]. It is well known that BRD4 regulates canonical stem cell markers like OCT4, SOX2, and c-MYC [119], which are upregulated in breast, epidermal (squamous carcinoma), and prostate cancer stem cells, promoting spheroid formation, aggressiveness and increased migrating potential [191, 192]. Importantly, BRD4 influences immunosuppressive transcription programs driven by OCT4/SOX2 and regulates additional transcriptional factors like ΔNp63α to sustain CSC phenotypes [193]. In glioma-initiating cells, BRD4 inhibition reduces self-renewal by modulating Notch1 signaling [194], while in MYC-driven medulloblastoma and prostate cancer, it impairs stemness by affecting transcription and mitochondrial function [195]. In esophageal adenocarcinoma (EAC), BRD4 controls cancer stemness by regulating Hippo/YAP1, a major regulator of cancer aggressiveness and cancer stemness [196]. The depletion of BRD4 affects YAP1 and subsequently restrains the CSC population and tumorigenesis. In breast cancer, the recognition of acetylated twist by BRD4 regulates the WNT65A expression and controls invasion, CSC-like properties, and tumorigenicity [197]. Additionally, BRD4 regulates MIR216A methylation, promoting stemness in gastric cancer [198]. These studies conclude that BRD4 is crucial in regulating CSCs in diverse tumor lineages.

In addition to BRD4, BPTF – an essential factor of embryonic development – has been implicated in cancer progression and stemness [199]. It enhances oncogenic signaling through c-MYC in melanoma and is positively associated with EMT markers in hepatocellular and colorectal carcinomas [199, 200]. In hepatocellular carcinoma, BPTF drives metastasis and stem-like traits by activating human telomerase reverse transcriptase (hTERT) expression [199], while its downregulation in ovarian cancer suppresses proliferation, migration, and colony formation [201]. Although BPTF is strongly linked to cancer stemness, its precise molecular mechanisms remain to be fully defined.

Beyond BET proteins, emerging evidence highlights the role of other non-BET proteins in regulating cancer. ATAD2 has emerged as a potential oncogenic target due to its elevated expression being associated with poor prognosis, enhanced invasiveness, and metastatic potential in cancers such as breast, ovarian, hepatocellular, and lung cancers [202–204]. Functional studies show that conditional deletion of ATAD2 reduces stem-like characteristics in lung cancer cells, while its silencing in esophageal squamous cell carcinoma (ESCC) disrupts Hedgehog signaling, induces apoptosis, and suppresses CSC proliferation, migration, and invasion – demonstrating its direct involvement in cancer stemness regulation [205, 206]. ATAD2 is frequently upregulated in high-grade tumors and modulates stemness-associated gene expression by activating E2F and c-MYC-driven transcriptional programs [207]. Moreover, m6A methylation enhances ATAD2 mRNA stability and facilitates its interaction with SOX9 in clear cell renal cell carcinoma (ccRCC), promoting enhancer assembly and the expression of CSC markers [208]. These findings underscore the critical role of ATAD2 in sustaining CSC properties across multiple malignancies.

Complementing the role of ATAD2, members of the BAZ protein family also exhibit significant but varied contributions to cancer biology. The BrD adjacent to the zinc finger (BAZ) protein family includes BAZ1A, BAZ1B, BAZ2A, and BAZ2B, each playing distinct roles in chromatin regulation and cancer biology [209]. BAZ1A, also known as ACF, promotes cellular senescence in both normal and malignant cells. Its downregulation leads to increased expression of the tumor suppressor p21 and decreased levels of proliferation markers such as Cyclin B2, Cyclin D1, and MKI67 [210]. BAZ1B contributes to chromatin organization, DNA repair, and cell cycle regulation and is implicated in oncogenesis, particularly in lung cancer, where its overexpression enhances proliferation, migration, and invasiveness [211]. In contrast, BAZ2 proteins exhibit context-dependent roles in cancer differentiation – BAZ2A is often linked with promoting cancer stemness, while BAZ2B appears to inhibit it [189]. Although these findings suggest a significant association between BAZ proteins and cancer stemness, detailed mechanistic insights remain limited.

In addition, KMT2A also plays a crucial role in gene dysregulation, malignant cell proliferation, and differentiation across various cancers, particularly hematological malignancies [212]. Its fusion proteins, generated through chromosomal translocations, are strongly associated with cancer development and the maintenance of CSC properties [187]. In leukemic stem cells, KMT2A fusions with genes like AF9, ENL, or ELL enhance self-renewal, block differentiation, and promote leukemogenesis through aberrant chromatin modification [212]. KMT2A regulates key genes such as *HOXA*, *MEIS1*, and *CDK6*, facilitating H3K4 methylation at their promoters to sustain active transcription [212, 213]. By maintaining open chromatin at regulatory sites, KMT2A supports stem cell maintenance, proliferation, and survival. Moreover, it engages with signaling pathways like Wnt/β-catenin and Notch to further promote CSC expansion and therapy resistance.

Similarly, SMARCA gene subgroup belongs to the SWI/SNF family and plays a critical role in transcription regulation, DNA repair, and stem cell maintenance [214]. Although SMARCA4 and SMARCA2 are traditionally considered tumor suppressors [215], their high expression has been associated with cancer progression and poor prognosis in several cancers [216]. SMARCA4, in particular, supports self-renewal and the maintenance of CSCs, especially in lung cancer, where its loss downregulates stemness genes and impairs tumor sphere formation [217]. Conversely, its overexpression is related to increased tumor formation, progression, and stem-like properties in lung cancer models [218]. Functioning as a chromatin remodeler, SMARCA4 modulates transcription by interacting with chromatin modifiers and transcription factors, influencing gene expression tied to proliferation, differentiation, and therapy resistance [219, 220]. In glioblastoma and ovarian cancer, SMARCA4 promotes epithelial-to-mesenchymal transition (EMT), metastasis, and resistance by enhancing histone acetylation and DNA repair gene expression, including TXNIP [221, 222]. Targeting SMARCA4 could sensitize CSCs to DNA-damaging agents and improve therapeutic outcomes.

SMARCA2 (BRM), closely related to SMARCA4, often compensates in SMARCA4-deficient cancers and exhibits context-dependent roles in CSC regulation [223]. In hepatocellular carcinoma, SMARCA2 promotes CSC maintenance and tumorigenesis, while in breast cancer, it appears to suppress self-renewal – opposite to SMARCA4 function [216]. SMARCA2 also regulates CSC behavior in prostate, colorectal, and melanoma cancers through pathways such as Wnt/β-catenin, and drives EMT, migration, and metastasis in pancreatic [224]. Like SMARCA4, SMARCA2 enhances DNA repair capabilities in ovarian cancer, contributing to therapeutic resistance [222]. Together, SMARCA2 and SMARCA4 govern CSC-associated traits such as stemness, metastasis, and treatment resistance across diverse malignancies, underscoring their significance as potential therapeutic targets in CSC-directed cancer therapies.

Likewise, TRIM24 plays a pivotal role in maintaining CSC self-renewal, stemness, and differentiation by regulating SOX2, a key transcription factor in cancers [225]. In glioblastoma, the elevated TRIM24 levels correlate with increased expression of these genes, enhancing CSC tumorigenicity. As a chromatin reader, TRIM24 binds H3K23ac-modified histones to activate stemness-related transcriptional programs, thereby supporting CSC plasticity and self-renewal. In breast cancer, TRIM24 interacts with ERα to dysregulate ERα target genes involved in proliferation and stemness, promoting CSC expansion and metastasis [226]. Knockdown of TRIM24 reduces tumor initiation in glioblastoma models, underscoring its oncogenic function [225]. Moreover, TRIM24 modulates epigenetic pathways such as Wnt/β-catenin, Hedgehog, and Notch; for instance, it stabilizes β-catenin in colorectal cancer to activate Wnt targets and enhance metastatic potential, and it promotes Notch signaling in breast cancer, contributing to therapeutic resistance and CSC proliferation [225, 227].

Moreover, other TRIM family members – including TRIM28, TRIM33, and TRIM66 – also play essential roles in maintaining CSC properties through epigenetic regulation [228]. TRIM28 supports CSC tumorigenicity in breast, glioblastoma, and liver cancers by modulating EMT-related genes and stemness markers such as CD133 and ALDH1 while repressing chromatin through histone-modifying enzymes to preserve self-renewal and an undifferentiated state [229]. TRIM33, via its PHD BrD, interacts with acetylated histones to regulate gene expression programs governing self-renewal and lineage differentiation [228, 230]. Functional loss of TRIM33 drives aggressive and therapy-resistant CSC phenotypes in a context-dependent manner across liver and breast cancers [228, 230]. TRIM25 enhances CSC characteristics in breast cancer by upregulating core stemness transcription factors (OCT4, SOX2, NANOG) and promoting resistance through anti-apoptotic signaling and DNA repair programs [231]. Collectively, these TRIM proteins underscore the broader significance of epigenetic modulation in CSC maintenance and highlight potential avenues for targeted therapies.

Additionally, p300, through its association with H3K27ac, plays a crucial role in regulating cancer progression and CSC properties [232]. H3K27ac is a hallmark of active enhancers, often linked to genes driven by oncogenic transcription factors [233]. In cancers such as gastric and breast, p300 binds H3K27ac-marked enhancers at loci *COL1A2* and *CRISP3*, contributing to drug resistance and CSC maintenance [234, 235]. Elevated *EP300* expression in triple-negative breast cancer (TNBC) enhances mesenchymal and stemness marker expression, supports anchorage-independent growth, and promotes chemoresistance [235]. Conversely, *EP300* knockdown reduces CSC populations, impairs migration, and diminishes invasive capacity [236]. These findings highlight the critical role of p300 in regulating CSC characteristics through its recognition of H3K27ac modifications across diverse cancer types.

Moreover, TAF1, a core component of the transcription factor IID (TFIID) complex, is important in initiating transcription and is implicated in various cancer-related pathways that influence cell survival, differentiation, and proliferation [237]. Its paralog, TAF1L, also contributes significantly to cancer biology [238]. Emerging studies suggest that TAF1L may support CSC maintenance and survival [239]. For instance, the IncRNA FOXD2-AS1 recruits TAF1 to the *NOTCH1* promoter, activating Notch signaling and promoting stemness while inhibiting apoptosis and differentiation in glioma cells. In esophageal squamous cell carcinoma, TAF1 overexpression enhances proliferation, migration, and invasion through modulation of the Akt pathway [238]. Similarly, TAF1L facilitates oral squamous cell carcinoma progression by inducing autophagy and aiding in apoptosis resistance [239]. However, a direct mechanistic link between these proteins and cancer stemness remains to be fully defined.

Upregulation of ASH1L has been associated with enhanced cancer cell proliferation, aggressive tumor behavior, and poor prognosis in breast, leukemia, liver, and thyroid cancers [81, 239–241]. In hepatocellular carcinoma specifically, elevated ASH1L levels promote proliferation, migration, and invasion [241]. Although ASH1L is known to influence stem cell differentiation, direct evidence linking it to cancer stemness is currently lacking. Similarly, while high expression of the histone acetyltransferase KAT2A has been correlated with cancer stemness in several solid tumors, the underlying mechanisms remain unclear [242]. The roles of epigenetic regulators such as BRD9 and CECR9 in CSC biology also remain poorly defined. Collectively, these findings underscore the critical role of chromatin-based regulatory mechanisms in maintaining CSC properties and suggest that targeting these pathways could offer promising strategies to modulate CSC phenotypes.

### Therapeutic Targeting of BrD Proteins in Cancer

Over the past decade, several research efforts have been made to develop selective BrD inhibitors [243]. A potent small molecule inhibitor should effectively dock deeply within the hydrophobic pocket of the BrD, competitively displacing Kac to disrupt histone interactions. Additionally, it must interact with the key conserved residue, asparagine, and engage with water molecules in a manner similar to its natural substrate, Kac [244]. Though the first BrD inhibitor, NPI, did not interact with the key residues, it effectively disrupted PCAF activity by blocking its association with HIV-1 F proteins – highlighting the potential of BrD inhibitors as therapeutic agents [15]. This finding encourages further research into developing BrD inhibitors capable of selectively displacing histone acetylation by targeting conserved residues. Subsequent discoveries led to the identification of potent and selective BET family inhibitors, particularly thienotriazolodiazepines [245]. These diazepine-based compounds, featuring thienodiazepine or hexodiazepine groups with a triazole ring, mimic Kac, dock deeply within the binding pocket, and interact with the surrounding hydrophobic residue, thereby enhancing their inhibitory effect [245]. Likewise, several studies demonstrate numerous selective inhibitors for both BET and non-BET BrD proteins (Tables 2 and 3). The following sections primarily focus on inhibitors that are effective against cancers and hold potential for clinical validation.

**Table 2:** BET inhibitors in clinical trials as monotherapy and with combination (https://clinicaltrials.gov). Most compounds listed are pan-BET inhibitors (unless otherwise specified) that target multiple BET family members (BRD2, BRD3, and BRD4)

Drug	Selective inhibition	Phase(s)	Combination therapies	Conditions
ABBV-075	pan-BET	I	Venetoclax; Bortezomib; Azacitidine	Breast cancer, non-small cell lung cancer, Acute myeloid leukemia, Multiple Myeloma, Prostate cancer, non-hodgkin’s lymphoma
ABBV-744	Selective BD2	I		Acute myeloid leukemia
BMS-986,158	pan-BET	I	Nivolumab; Ruxolitinib; Fedratinib	Pediatric Cancer
BMS-986,378	pan-BET	I	Cisplatin; Etoposide; Carboplatin; Nivolumab	Pediatric Cancer, Astrocytoma, Glioblastoma, Non-hodgkin’s lymphoma
CPI-0610	pan-BET	I/II	Ruxolitinib	Multiple Myeloma, Acute Leukemia
I-BET762	pan-BET	I/II	Fulvestrant; Entinostat; Trametinib; Cisplatin; Rifampicin	Acute myeloid leukemia, Multiple Myeloma, Non-hodgkin’s lymphoma and NUT midline carcinoma
OTX015	pan-BET	I/II	Azacitidine	Hematologic Malignancies, pancreatic ductal adenocarcinoma and Acute myeloid leukemia
RO6870810	pan-BET	I/II	Entinostat; Trametinib; Venetoclax; Atezolizumab; Daratumumab	Solid Tumors, Advanced Solid Tumors and Multiple Myeloma
SYHA1801	-	I		Advanced Solid Tumors
TQB3617	pan-BET	I		Advanced Malignant Tumors
ZEN-3694	pan-BET	I/Ib/II	Talazoparib; Pembrolizumab; Nab-paclitaxel; Binimetinib; Cisplatin; Abemaciclib	Metastatic castration-resistant prostate cancer, Squamous Cell Lung Cancer, and Solid Tumor
EP31670	dual BET and CBP/p300	I		Solid Tumor NUT Carcinoma
MK-8628	pan-BET	1/II		NMC Solid Tumor
CC-95,775	pan-BET	Ib		Lymphoma
FT-1101	pan-BET	I/Ib		Acute myeloid lukemia
GSK525762	pan-BET	I/II		Hematological Malignancies, and NUT
INCB054329	pan-BET	I/II		Solid Tumors, Hematologic malignancy
iBET 151	pan-BET	I	SGC0946; Cisplatin	
JQ1	pan-BET		5-fluorouracil or oxaliplatin	Colorectal cancer
NHWD-870	pan-BET	I		Melanoma; Osteosarcoma

**Table 3:** Summary of non-BET BrD inhibitors evaluated in in vivo studies

BrD family	Selective BrD Inhibitors	Type of cancer	References
ASH1L	AS99	Leukemia	[246]
ATAD2	AM879, AZ13824347, GSK8814, BAY-850	Breast cancer	[247–250]
BAZ1A/B	Cpd-2; BAZ1A-IN-1	Head and neck squamous cell carcinoma; leukemia, lung and breast cancer	[251]
BAZ2A/B	BAZ2-ICR; GSK2801		[252, 253]
BPTF	AU1, TP-238, GSK4027, DCB29, C620-0696, BZ1	Lung cancer and breast cancer	[254, 255], 256– [257]
BRD8	DN01 and DN02		
BRD7/9	I-BRD9, LP99, BI-7273, BI-7189, TP-472 and BI-9564	AML and Melanoma	[258–260]
BRPF1/3	IACS-9571; GSK685; NI-42 and BAY-299		
CECR2	NVS-CECR2-1; and DC-CBi-22	Colon cancer	[261]
CBP	MS2126, MS7972; Ischemin, SGC-CBP30, PF-CBP1, CPI-637, I-CBP112 and CPI-637	Leukemia	[262–265]
EP300	1-(Indolizin-3-yl) ethan-1-one	Prostate cancer	
KAT2A/2B	AUTX703	AML	[242–339]
SMARCA2/4 and PB1	PFI-3	AML, Lung cancer and rhabdoid	
TAF1/TAF1L	BAY-299	Acute myeloid lukemia	
TRIM24	IACS-9571 and N-benzyl-3,6-dimethylbenzo[d]- isoxazol-5- amine derivatives	non-small cell lung cancer	
ZMYND8	iZMYND8-34	Prostate cancer	[340]

### BET Inhibitors

BET inhibitors are potential targeted interventions in clinical development that control epigenetic regulation in cancer therapies [268]. BET proteins, particularly BRD2 and BRD4, are associated with several cancers by promoting oncogene expression [268]. Small molecules targeting these BET proteins have emerged as a potential target for cancer therapeutics. BET inhibitors are categorized into pan/non-selective BET inhibitors, selective BET inhibitors, and BET degraders. All these inhibitors displace BET proteins from enhancer regions, thereby controlling cancer cell progression. The potential of BET inhibitors in tumor suppression was first demonstrated in 2008, with subsequent studies in 2010 highlighting their anti-tumor and anti-inflammatory effects [190, 269, 270]. Since then, numerous investigations have demonstrated the significance of BET inhibitors across a range of solid and hematological malignancies (Table 2).

JQ1, a first-in-class pan BET inhibitor, competitively binds to the BET BrD pocket that recognizes Kac. It significantly reduces the tumor burden in hematological malignancies and NUT midline carcinoma by inducing apoptosis and suppressing MYC-driven transcription [271–273]. Despite being a promising inhibitor, the emergence of JQ1 resistance, off-target toxicity, and poor bioavailability have limited its clinical relevance [274]. To address these limitations, OTX015, a structurally similar analog of JQ1, was developed with improved pharmacological properties [275]. OTX015 inhibits all three BET proteins except BRDT and has demonstrated superior anti-tumor potential against multiple myeloma, B-cell lymphoma, and neuroblastoma [274, 276]. Another pan-BET inhibitor, I-BET762, is a synthetic small molecule that mimics Kac and disrupts chromatin complexes essential for oncogene transcription [19]. It has shown efficacy across various cancer cell types, including breast, lung, and gastrointestinal cancers. However, the adverse effects of these pan-BET inhibitors mask their relevance to clinical applications.

Selective BD1 and BD2 inhibitors are currently being studied to mitigate the adverse effects of pan-BET inhibition. Recent studies strongly suggest that targeting a single BrD of BET proteins may be sufficient to suppress cancer progression. Compounds such as MS436, Olinone, and GSK789 selectively inhibit BD1 and block BET function [274]. Notably, ABBV-075 (also known as Mivebresib), a selective BD2 inhibitor, has shown promising antitumor activity in both hematologic malignancies and solid tumors by disrupting chromatin complexes and downregulating *MYC* expression, ultimately inducing cancer cell death [277]. However, studies showcase contrasting evidence regarding the selective inhibition of BD1 or BD2 in replicating the therapeutic effects of pan-BET inhibitors [278]. While some studies suggest that BD1 inhibition exhibits antitumor effects while others propose that BD2 inhibition is related to anti-inflammatory activity [277–280]. These findings highlight the diverse functions of BET proteins, which may vary depending on tissue type and disease context.

### Non-BET Inhibitors

As discussed earlier, the HAT family members are major regulators of cancer progression, controlling critical cellular events such as cell cycle, DNA repair mechanisms, and gene expression. Among HATS, p300/CBP function has often been associated with hematologic malignancies and other solid tumors [281]. CBP BrD inhibitors represent the most extensively studied group after BET inhibitors. I-CBP112 was the first successful CBP and p300 BrD inhibitor that impairs the self-renewal capacity of leukemia [262]. Currently, CCS1477 and A-485 are potent and selectively targets BrD of p300/CBP. CCS1477 binds to the p300/CBP BrD, inducing cell-cycle arrest and differentiation in hematological malignancies. In contrast, A-485 inhibits HAT activity by blocking histone acetylation [282, 283], demonstrating high efficacy against hematological malignancies and androgen receptor (AR)-driven cancers. Another inhibitor, C646, competitively binds to the acetyl-CoA binding site of p300/CBP and inhibits cancer cell proliferation in pancreatic cancer, melanoma, leukemia, and breast cancers [284]. However, despite its broad activity, C646 exhibits relatively limited selectivity and potency over other inhibitors [282]. Both CCS1477 and A-485 have shown promising antitumor activity in diverse cancer models, including prostate cancer and hematologic malignancies. In addition to BET inhibitors, several selective non-BET BrD inhibitors have been identified, as summarized in Table 3.

In parallel, ATAD2 has emerged as an important epigenetic regulator involved in chromatin remodeling. It recognizes Kac on histones via its BrD and modulates chromatin accessibility. Structurally, the N-terminal region of ATAD2 anchors it to chromatin, while the AAA + ATPase domain acts as a molecular motor, facilitating nucleosome remodeling and enabling the BrD domain to recognize Kac sites on histone [110]. This coordinated activity enhances chromatin accessibility and promotes transcriptional programs that drive cancer progression, particularly in melanoma [285]. Inhibition of ATAD2 preferentially disrupts super-enhancers, leading to the selective downregulation of oncogenic drivers in CSCs. Notably, ATAD2 serves as a molecular bridge between methionine metabolism and super-enhancer assembly, highlighting its therapeutic potential as a CSC-targeted strategy [208].

Recent advances by both academic and pharmaceutical groups have highlighted ATAD2 as a promising therapeutic target in cancer, particularly through the development of specific inhibitors that disrupt its interaction with Kac residues. Apirat Chaikuad et al. (2014) first identified ATAD2 inhibitors using fragment-based screening, leading to nine novel hits, including thymidine and 3-methylquinolin-2(1 H)-one [286]. The crystal structure of ligand-bound ATAD2 revealed two conserved hydrogen bonds between the endylamide group of the ligand and ASN1064 residue, a critical residue located in the substrate-binding pocket that directly interacts with Kac [90, 286]. Additionally, a methyl group adjacent to the binding core further stabilizes the interactions through non-covalent bonding with neighboring residues. These structural insights positioned 3-methylquinolin-2(1 H)-one as a promising ATAD2 inhibitor that mimics acetyl-lysine and provides a starting scaffold for developing highly potent inhibitors. Further structure-based study led by John E. Ladbury revealed an unusual ligand-induced conformational change involving the conserved ASN1064 residue [287]. Notably, the discovery of multiple orientations of key gatekeeper residues, such as Ile1074, Val1008, and Val1013, upon ligand binding fosters the plasticity of the selective ATAD2 binding pocket [287]. These dynamic interactions promote ZA-loop rigidification and hydrophobic side chain rearrangements, providing a structural basis for the rational design of selective and potent ATAD2 inhibitors.

Building on these findings, AstraZeneca subsequently discovered two novel classes of ATAD2 inhibitors, namely azepines and imidazolidine-2,4-dione derivatives, which demonstrated sub-micromolar IC50 values, indicating strong inhibitor activity [247]. Shortly after, Bayer advanced the field by developing imidazolidine-2,4-dione and furan-based compounds, culminating in the identification of BAY-850 – a selective ATAD2 inhibitor that exploits atypical binding modes (patent WO2017093272A1). Among these, an isoform of a formylamine derivative and a cyclohexane-1,4-diamine analog, also designated as BAY-850, exhibited enhanced potency with nanomolar-range IC50 values [248]. Despite these promising developments, the unique spatial architecture of the ATAD2 BrD continues to pose challenges, and further efforts are needed to optimize small-molecule inhibitors for clinical applications.

In parallel with efforts to inhibit ATAD2, several inhibitors targeting other BrD-containing proteins, such as BRPF family members, have been developed and are under active investigation for their therapeutic potential in cancer. These Compounds are designated to specifically target the BrDs of BRPF proteins, thereby preventing their recognition of Kac residues on histones and disrupting the activity of the MOZ/MORF (monocytic leukemic zinc-finger protein complex and the MOZ-related factor) HAT complexes. Among these, GSK5959 has emerged as a selective BRPF inhibitor with promising anti-proliferative effects in leukemia, liver cancer, and breast cancer cell lines [262, 288]. Notably, GSK5959 treatment suppresses the expression of key stemness-associated genes, including *NOTCH1*, *OCT4*, and *EPCAM*, thereby limiting CSC progression in liver cancer. Another compound, BAY-299 is a potent dual inhibitor targeting both BRPF2 and TAF1, which has demonstrated significant anti-tumor activity in preclinical models of AML and TNBCs [289]. In addition to reducing tumor growth and enhancing the effects of chemotherapeutic agents, BAY-299 has been shown to induce a tumor-suppressive immune response in specific TNBC subsets, providing a dual mechanism for impairing tumor progression [237, 289, 290].

Further expanding the scope of selective non-BET BrD inhibitors, GSK2801 has been developed to target the BrDs of BAZ2A/B and BRD9. Chemical knockdown of BAZ2A/B using GSK2801, particularly in combination with the pan-BET inhibitor JQ1, selectively displaces BRD2 from super-enhancers associated with ETS-regulated genes. This combinatorial inhibition effectively blocks BRD2-driven transcriptional programs, leading to apoptosis in TNBCs [291]. Another selective inhibitor, I-BRD9, specifically targets the BRD9 BrD [292] and has demonstrated antiproliferative effects in several malignancies, including AML, gallbladder cancer, colon cancer, and clear cell renal cell carcinomas (ccRCCs) [293–296]. Interestingly, in ccRCC models, I-BRD9 suppresses tumor growth in HIF2α^low/−^ cells but is ineffective in HIF2α^high^ contexts, suggesting a dependency on hypoxia signaling states [296]. Notably, I-BRD9 has also been shown to inhibit the growth of rhabdoid tumors, a class of highly aggressive pediatric malignancies [296], by inducing cell cycle arrest and apoptosis, particularly in AML and rhabdoid tumor models [292, 293].

Moreover, small molecule inhibitors targeting the BrDs of the SWI/SNF chromatin remodeling complex (Family VIII) are under active development for cancer therapeutics [297]. PFI-3 was the first selective inhibitor of SMARCA4, SMARCA2, and PBRM1 BrDs [298]. In multiple myeloma, PFI-3 displaces SMARCA2 and the oncogenic driver NSD from chromatin, leading to suppression of oncogene transcription and tumor cell proliferation [299]. Interestingly, PFI-3 also enhances the anti-tumor efficacy of temozolomide and sensitizes drug-resistant glioblastoma cells to treatment [300]. These findings highlight the clinical relevance of targeting SWI/SNF BrDs, and next-generation inhibitors are currently underway to improve cellular potency and therapeutic impact.

Additionally, several SET domain-containing HMT inhibitors are in preclinical and clinical development for cancer therapy. One notable example is Tazemostat, a selective EZH2 inhibitor that has shown promising efficacy in clinical trials, particularly for treating follicular lymphoma. Another selective EZH2 Inhibitor, GSK126, demonstrates robust anti-cancer activity in preclinical models by inhibiting cell proliferation and inducing apoptosis [301]. Beyond EZH2, UNC0638 is a potent and selective inhibitor of G9a and GLP (G9a-like protein) that effectively reduces H3K9me2 marks on chromatin. In preclinical models of lung and breast cancer, UNC0638 reactivates silenced tumor suppressor genes and inhibits cancer cell proliferation [302]. Similarly, BIX-01294, another G9a inhibitor, also reduces H3K9me2 levels and induces re-expression of epigenetically silenced genes. It has demonstrated efficacy in suppressing tumor growth and enhancing the effects of chemotherapy in preclinical models of prostate and ovarian cancer [303].

Furthermore, Bromosporine was the first small molecule inhibitor identified to target BrD PHD finger transcription factor (BPTF), showing activity in the sub-micromolar range [304]. Originally considered as a pan-BET inhibitor with nanomolar potency, Bromosporine exhibited only moderate affinity for BPTF. Subsequent screening efforts by Urick et al. led to the identification of AU1, a more potent selective BPTF inhibitor, from a library of 229 small molecules [254]. AU1 was found to reduce cell proliferation, induce G1 cell cycle arrest, and diminish c-MYC-DNA binding, indicating its impact on oncogenic transcriptional programs [305]. Structure-activity relationship (SAR) studies further optimized AU1, leading to the identification of its S-enantiomer, which showed enhanced potency against leukemia cells [306]. Despite being one of the few known BPTF inhibitors, AU1 faces several limitations, including chemical instability, off-target toxicity, and limited in vivo efficiency. Other compounds, such as DCB29 and C620-0696 [255], have also been reported to bind BPTF and exhibit moderate anticancer effects; however, detailed analysis of their mechanisms and therapeutic potential remains lacking.

Given that many BrD proteins are critical for maintaining transcriptional networks in normal stem and progenitor cells, broad BrD inhibition can undesirably disrupt self-renewal and tissue homeostasis [119, 177, 178, 307]. To mitigate such toxicity, recent studies emphasize domain selectivity, context-specific pharmacology, and targeted delivery approaches. Within the BET family, BD1- or BD2-selctive inhibitors such as ABBV-744 and ABBV-075 (Mivebresib) maintain potent antitumor activity while exhibiting improved tolerability compared to pan-BET inhibitors [280, 307]. PROTAC-based degraders (MZ1, ARV-771) further enable transient and reversible BET modulation that spares normal hematopoietic compartments [307–309]. Beyond BET proteins, selective CBP/p300 inhibitors (A-485, CCS1477) induce differentiation and cell cycle arrest in tumor cells while limiting global transcription perturbation [282, 310]. Likewise, non-BET BrD inhibitors such as I-BRD9 for BRD9 [292], PFI-3 for SMARCA2/4/PBRM1 ([311]), and BAY-850 for ATAD2 ([248]) demonstrate context-specific efficacy in malignancies driven by SWI/SNF or chromatin-remodeling dependencies. Complementary strategies such as nanoparticle- or antibody-guided delivery and intermittent or low-dose regimens further reduce systemic exposure and off-target transcriptional stress [268]. Taken together these advances support that BrD remain actionable cancer targets; however, optimal therapeutic benefit requires domain/family selectivity and pharmacologic fine-tuning to preserve normal stem-cell function while maintaining antitumor efficacy.

### BrD Protein Inhibition To Overcome Drug Resistance

Monotherapy remains a common treatment strategy for various cancers; however, it is frequently challenged by the emergence of drug-resistant cancer cells [312, 313]. In addition, chemotherapy administered as a single agent often fails to eliminate CSCs within the tumor microenvironment, which can further promote self-renewal, differentiation, and invasiveness [314]. As a result, combinatorial therapies targeting multiple oncogenic pathways have become a cornerstone of modern cancer treatment. While several targeted inhibitors show promise, their efficacy as monotherapies is limited. This has prompted efforts to explore BrD inhibitors in combination with other agents to enhance therapeutic outcomes and overcome resistance [315]. Notably, BrD inhibitors have been evaluated alongside kinase inhibitors, Bcl-2 inhibitors, HMT inhibitors, HDAC inhibitors, and conventional chemotherapeutics. Agents such as cisplatin and paclitaxel have been used in combination regimens across various advanced cancers [316]. For example, combining BET inhibitors with cisplatin or paclitaxel synergistically suppresses tumor cell growth in NSCLC [317]. This combinatorial effect is mediated through the inhibition of autophagy and induction of apoptosis.

Several studies have demonstrated that combining BET inhibitors such as JQ1, OTX015, RVX2135, and I-BET151 with HDAC inhibitors leads to enhanced tumor suppression [318, 319]. For instance, co-treatment with JQ1 or RVX2135 and SAHA (a pan-HDAC inhibitor) significantly increases apoptosis in lymphoma cells compared to either agent alone. Notably, the combination of RVX2135 and SAHA not only inhibits tumor growth but also reduces white blood cell counts – effects not observed with monotherapy. Similarly, treatment with JQ1 in combination with other HDAC inhibitors such as vorinostat, sodium butyrate, and trichostatin A (TSA) effectively suppresses cell viability in OCI-AML cells. Beyond HDAC inhibitors, combining BET inhibitors with other agents has shown therapeutic promise. In AML, co-treatment with venetoclax (a BCL2 inhibitor) and ABBV-075 promotes apoptosis by downregulating *MCL1* and *Bcl-xL* levels [320, 321]. In ovarian cancer, the BET inhibitor AZD5153 has been shown to reverse resistance to the PARP inhibitor olaparib by downregulating *PTEN*, thereby sensitizing tumor cells to treatment [322].

Likewise, Bruton’s tyrosine kinase (BTK) dysregulation is frequently associated with B-cell malignancies [323]. Targeted inhibitors such as AVL-292, ibrutinib, and ONO-4059 have shown efficacy in controlling these cancers. However, prolonged use of these agents often leads to drug resistance and disease relapse. Recent studies have demonstrated that the BET inhibitor OTX015 exhibits synergistic effects when combined with everolimus or ibrutinib in mature B-cell lymphoid malignancies [276, 324]. Moreover, OTX015 combined with BEZ235 shows promising activity against ibrutinib-resistant mantle cell lymphoma (MCL) cells [325].

Expanding beyond hematologic malignancies, BET inhibitors also show strong synergy with kinase inhibitors in solid tumors, particularly breast cancers. In both HER2 + and TNBC models, JQ1 enhances the durability of kinase inhibitors such as lapatinib and trametinib, resulting in sustained tumor growth inhibition in vitro and in vivo [326]. Mechanistically, JQ1 blocks enhancer remodeling and inhibits p-TEFb-dependent transcriptional reprogramming, thereby preventing the adaptive resistance typically induced by RTK and MEK-ERK pathway inhibition [326, 327]. High-throughput screens in TNBC models have identified additional synergistic partners for BET inhibition, including MEK1/2, CDK9, p300/CBP, and Aurora kinase inhibitors [327, 328]. Notably, combinations with the microtubule poison paclitaxel or the AXL kinase inhibitor TP0903 also synergize with JQ1 to reduce proliferation in SUM149R and SUM159R TNBC models [328]. Among all tested combinations, the CDK4/6 inhibitor palbociclib demonstrated the most robust synergy with JQ1 across parental, drug-resistant, and additional TNBC lines, irrespective of RB1 mutation status [328]. In broader breast cancer models, targeting BET BrDs through genetic or pharmacological means reduces the expression of resistance-associated kinases induced by lapatinib, including ERBB3, IGF1R, DDR1, MET, and FGFRs, thereby disrupting SRC/FAK-AKT signaling [329]. When used alongside kinase inhibitors, this strategy limits adaptive kinome shifts and strengthens the durability of the therapeutic response.

Emerging evidence suggest that BET proteins are involved in modulating the PD1/PD-L1 immune checkpoint axis, and their inhibition can enhance the therapeutic impact of checkpoint blockade [52]. BRD4, in particular, has been shown to promote PD-L1 expression by binding to the CD274 promoter and facilitating its transcription [53]. In an ID8-Defb29/Vegf-a syngeneic overian cancer model, the BET inhibitor JQ1 reduced tumor growth, lowered PD-L1 levels on both tumor and immune cells and enhanced cytotoxic T cell function [52]. In KRAS-mutant NSCLC xenografts, combining JQ1 with a PD-1 inhibitor produced synergistic reductions in tumor burden and extended survival. Likewise, the clinical-stage BET inhibitor INCB054329 demonstrated synergistic antitumor activity when paired with PD1 or PD-L1 blockade across several syngeneic murine tumor models [53]. This synergism was also observed when INCB054329 was combined with the IDO-1 inhibitor epacadostat, suggesting that BET proteins may influence multiple immune checkpoint pathways.

In TNBC, co-targeting BET (BRD4) and non-BET (BAZ2/BRD9) BrDs using JQ1 and GSK2801 effectively suppresses BRD2-driven transcription and enhances growth inhibition in both 2D and 3D cultures [291]. Interestingly, the mode of cell death appears context-dependent; senescence dominates in 2D cultures, whereas apoptosis is more pronounced in 3D spheroids, likely due to differences in cell adhesion and intercellular interactions. BrD inhibition has also shown promise in other cancers by enhancing therapeutic efficiency and overcoming resistance. In pancreatic ductal adenocarcinoma, the combination of the BrD inhibitor JQ1 and the neddylation inhibitor MLN4924 synergistically reduces cell growth and viability by increasing reactive oxygen species (ROS), triggering DNA damage and apoptosis, highlighting its potential for clinical development [330]. Similarly, in KMT2A-rearranged leukemia, the combination of the second-generation BET inhibitor ABBV-744 with the GSK3 inhibitor CHIR-98,014 effectively suppressed disease progression in patient-derived xenograft models [331]. This therapeutic strategy was guided by CRISPR-based loss-of-function screens identifying speckle-type POZ protein deficiency as a key mediator of BET inhibitor resistance, along with proteomic and kinase vulnerability analyses, which showed increased sensitivity to GSK3 inhibition in BET inhibitor-treated cells.

In a phase Ib/IIa study, the combination of the BET inhibitor ZEN-3694 with enzalutamide demonstrated manageable safety and effective target inhibition in patients with metastatic castration-resistant prostate cancer [332]. This treatment extended radiographic progression-free survival to nine months and may help re-sensitize tumors with low androgen receptor activity. In multiple myeloma, inhibition of BRD9 using the degrader QA-68 enhanced the efficacy of immunomodulatory drug (IMiD) pomalidomide by downregulating *MYC* and upregulating *CRBN*, resulting in a significant reduction of tumor cell growth [333]. Notably, BRD9 targeting also re-sensitizes resistant cells to the next-generation IMiD iberdomide, highlighting the potential of dual inhibition of BRD9 and IKZF3 as a promising approach for treating drug-resistant myeloma [333]. In melanoma, the combination of bromosporine (a BrD inhibitor) and cobimetinib (a MEK inhibitor) showed potent anti-tumor activity in BRAFi-resistant, treatment-naive BRAF mutant, and immunotherapy-resistant NRAS- and NF1-mutant subtypes. This synergistic effect was driven by inducing DNA damage, downregulating mitotic genes, and promoting cell-cycle arrest and apoptosis [334].

Similarly, in hematologic malignancies (particularly multiple myeloma), proteasome inhibitors such as bortezomib, ixabomib, and carfilzomib remain foundational therapies. Yet, resistance to these agents remains a major clinical challenge, promoting interest in alternative treatment strategies. In this context, BET inhibitors, including JQ1, I-BET151, and I-BET762, have been investigated for their potential in both hematologic and solid tumors. Combinatorial regimens pairing BET inhibitors with carfilzomib have shown synergistic anti-cancer activity [335], while the JQ1 analog, CPI-203, in combination with lenalidomide, demonstrated efficacy in bortezomib-resistant myeloma cells [336]. Although BrD inhibitors typically exhibit low off-target toxicity as monotherapies, their therapeutic impact is most pronounced when used in rational combinations with other targeted agents. Altogether, BrD inhibitors represent a versatile and clinically relevant class of compounds with strong potential for integration into next-generation cancer treatment regimens.

## Conclusion

In summary, BrD-containing proteins have emerged as indispensable regulators at the intersection of chromatin biology, stem cell function, and cancer. These evolutionarily conserved epigenetic readers interpret histone acetylation marks to modulate gene expression programs that define stem cell identity, lineage specification, and CSCs maintenance. This review provided a comprehensive overview of the structural and functional diversity of the BrD protein family, emphasizing their roles in regulating pluripotent and multipotent stem cell states and their dysregulation in cancers. Members of this family include coactivators such as p300/CBP and subunits of chromatin remodeling complexes such as SWI/SNF (e.g., BRD7, BRD9), ISWI, and MYST HATs, which interact with master transcription factors (OCT4, SOX2, NANOG, and MYC) to orchestrate both normal and malignant stem cell programs. While members of the BET family, particularly BRD4, have been extensively characterized for their enhancer-associated transcriptional regulation and stemness maintenance, non-BET BrDs such as ATAD2, BPTF, SMARCA4, and TRIM family members also promote CSC phenotypes, driving heterogeneity, metastasis, and therapy resistance. Notably, the development of small-molecule BrD inhibitors, especially those targeting BET domains, has demonstrated significant potential in disrupting oncogenic transcription, depleting CSC populations, and enhancing therapy response. BrD-targeted therapies are also emerging as promising strategies to overcome drug resistance in cancer. However, their clinical utility is constrained by limited selectivity, feedback resistance, and context-dependent BrD activity across tissues and tumor types.

To fully harness the therapeutic potential of BrD proteins, further investigation is needed, particularly into the poorly understood roles of non-BET BrDs in stemness and CSC biology. Exploring their interactions with non-histone substrates, noncoding RNAs, and 3D chromatin architecture will provide deeper insights into how they shape transcriptional programs. Given functional overlap within the BrD family, defining their context-specific roles across stem cell types and tumor lineage is essential to minimize off-target effects. Their ability to integrate extracellular signals with chromatin state changes, particularly through enhancer-promoter communication and chromatin looping, suggests a broader role in dynamic transcriptional reprogramming during differentiation and tumor evolution. Identifying CSC-specific BrD expression patterns and dependencies may enable precision targeting, while advances in single-cell and spatial multi-omics will deepen insights into BrD function within heterogeneous and spatially restricted CSC niches. Moreover, combining BrD inhibition with other therapeutic modalities, such as DNA damage response inhibitors, immune checkpoint blockade, or other epigenetic modulators, may synergistically eliminate resistant CSCs and prevent tumor relapse. Taken together, BrD proteins are key regulators of stem cell and cancer biology, and their dual role in shaping both the transcriptome and epigenome makes them promising molecular targets for regenerative medicine and precision oncology.

## Data Availability

No datasets were generated or analysed during the current study.

## Declarations

### Clinical Trial Registration

N/A.

### Permission To Reproduce Materials from Other Sources

N/A.
